# Microsphere Strategy to Generate Conformal Bone Organoid Units with Osteoimmunomodulation and Sustainable Oxygen Supply for Bone Regeneration

**DOI:** 10.1002/advs.202501437

**Published:** 2025-06-20

**Authors:** Anfu Deng, Hao Zhang, Yan Hu, Jilong Li, Jian Wang, Xiao Chen, Zhen Geng, Ke Xu, Yuxiao Lai, Jianhua Wang, Yingying Jing, Long Bai, Jiacan Su

**Affiliations:** ^1^ Organoid Research Center Institute of Translational Medicine Shanghai University Shanghai 200444 China; ^2^ MedEng‐X Institutes Shanghai University Shanghai 200444 China; ^3^ National Center for Translational Medicine (Shanghai) SHU Branch Shanghai University Shanghai 200444 China; ^4^ School of Medicine Shanghai University Shanghai 200444 China; ^5^ Department of Orthopedics Xinhua Hospital Affiliated to Shanghai Jiao Tong University School of Medicine Shanghai 200092 China; ^6^ Translational Medicine R&D Center Institute of Biomedical and Health Engineering Shenzhen Institute of Advanced Technology Chinese Academy of Sciences Shenzhen 518055 China; ^7^ University of Chinese Academy of Sciences Beijing 100049 China; ^8^ Wenzhou Institute of Shanghai University Wenzhou Zhejiang 325000 China

**Keywords:** bone organoid, bone regeneration, macrophage, osteoimmunomodulation, oxygen release

## Abstract

Repairing large bone defects remains a major challenge, as current strategies—including autografts, allografts, and tissue‐engineered scaffolds—are limited by donor shortage, suboptimal biocompatibility, and insufficient control of the regenerative microenvironment. Bone organoids offer a promising strategy by simulating bone tissue functions and microenvironments in vitro, showing great potential for bone repair. However, current bone organoids still face issues such as insufficient oxygen supply, and limited immune modulation, reducing their effectiveness for complex bone defect repair, especially when precise shape conformity is required. To address these challenges, bone organoid unit based on silk hydrogel microspheres is developed, introducing oxygen‐releasing components and immune cells to better mimic the bone repair environment and enable conformal filling of irregular defects. In vitro, the system formed bone organoid units through 28 days culture that sustained cell viability, promoted M2 macrophage polarization, and enhanced osteogenesis and angiogenesis. In a critical‐sized mouse cranial defect model, this strategy outperformed traditional approaches, showing superior bone regeneration and tissue integration. These findings indicate that the strategy effectively addresses key obstacles in bone repair, such as oxygen supply, immune modulation, and shape conformity, providing a promising solution for personalized bone regeneration.

## Graphical abstract

Oxygen‐releasing immunomodulatory microspheres are used to construct conformal bone organoid units that enhance osteogenesis, angiogenesis, and immune regulation. By integrating BMSCs, macrophages, and CaO₂‐HAp into a SilMA‐based hydrogel platform, this strategy provides sustained oxygen supply and anti‐inflammatory microenvironments, leading to effective bone regeneration and personalized repair of critical‐sized bone defects.

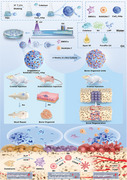

## 1 Introduction

Critical‐size bone defects, caused by trauma, tumor resection, or osteoporosis, are often associated with prolonged recovery periods and functional impairments, which can increase the risk of nonunion.^[^ ^1^ ^]^ These defects remain a major challenge in orthopedic clinical practice and a prominent research focus in regenerative medicine. While traditional methods of bone repair, such as metal implants, autologous bone grafts, and allografts, provide partial solutions, each comes with significant limitations.^[^ ^2^ ^]^ Metal implants offer mechanical stability but struggle to integrate well with surrounding bone tissue, leading to complications such as infection and poor bone integration.^[^ ^3^ ^]^ Autologous and allogeneic bone grafts, while biologically active, face challenges related to donor site morbidity,^[^ ^4^ ^]^ immune rejection,^[^ ^5^ ^]^ and limited material availability,^[^ ^6^ ^]^ particularly when addressing large‐scale bone defects.^[^ ^7^ ^]^ Therefore, there is an urgent need for more efficient, flexible, and personalized bone repair strategies that can promote bone regeneration, especially for critical‐sized bone defects.

With the advancement of regenerative medicine, organoid technology has emerged as a promising solution and has garnered significant attention.^[^ ^8^ ^]^ Organoids are 3D, in vitro reconstructed miniature organs that replicate the structure and function of human tissues, significantly advancing tissue regeneration research. Recent progress in soft tissue organoids, such as liver, neural, and intestinal organoids, has opened new possibilities for disease modeling and treatment.^[^ ^9^ ^]^ These soft tissue organoids have demonstrated great potential in regenerative medicine, particularly in drug screening, disease research, and tissue repair.^[^ ^10^ ^]^ However, constructing bone tissue, a hard tissue, presents unique challenges that differ significantly from soft tissues. Bone tissue requires not only mechanical support and load‐bearing capacity but also high biological activity, including osteogenesis and angiogenesis.^[^ ^11^ ^]^ Traditional organoid construction typically relies on natural matrix gels (such as Matrigel) extracted from mice, but these materials have several drawbacks, including high cost, undefined composition, batch‐to‐batch variability, and high immunogenicity.^[^ ^12^ ^]^ Additionally, their mechanical properties are insufficient to replicate the mechanical characteristics of bone tissue. Recent advances in biomaterials have prompted researchers to explore more suitable alternatives, such as silk fibroin (SilMA) hydrogels, gelatin, and polylactic acid, which offer unique advantages in biocompatibility, mechanical properties, and versatility, providing novel strategies for bone organoid construction.^[^ ^13^ ^]^ SilMA is a photocrosslinkable hydrogel derived from natural silk fibroin, widely recognized for its biocompatibility, mechanical tunability, and mild gelation conditions. It enables the formation of stable, cell‐friendly 3D scaffolds that support the survival and differentiation of various cell types. SilMA also exhibits good permeability to nutrients and oxygen, and its degradation products‐amino acids, peptides, and low‐molecular‐weight proteins are naturally metabolized in vivo, reducing cytotoxicity and inflammation risks. These features make SilMA an ideal base material for constructing bone organoids, providing both structural support and a favorable microenvironment for osteogenesis and immune regulation.^[^ ^14^ ^]^

Building on previous work in bone organoid engineering, including the use of HAp‐based hybrid bioinks,^[^ ^15^ ^]^ dynamic DNA‐hydrogel systems,^[^ ^16^ ^]^ and ECM‐DNA‐CPO‐based bionic matrices^[^ ^17^ ^]^ to support osteogenic differentiation and spatial organization, our team has demonstrated the feasibility of constructing large‐scale bone‐like tissue in vitro and applying it to bone defect models with promising outcomes. However, significant limitations remain that hinder the translational potential of current bone organoid models. One major challenge is the lack of a sufficient oxygen supply during long‐term in vitro culture and after implantation. Bone is a highly vascularized tissue, and oxygen is essential for maintaining cell viability, promoting osteogenic differentiation, and supporting extracellular matrix mineralization.^[^ ^18^ ^]^ Most bone organoid constructs to date do not incorporate functional vasculature or oxygen delivery systems, resulting in hypoxic microenvironments that impair tissue maturation and regenerative capacity.^[^ ^19^ ^]^ Another key limitation is the absence of immune components. Recent studies have highlighted the essential role of macrophage‐mediated immune modulation in bone healing, particularly the polarization toward the M2 phenotype, which supports angiogenesis and suppresses inflammation.^[^ ^20^ ^]^ However, current bone organoids typically exclude immune‐regulatory elements, overlooking their synergistic role in orchestrating tissue regeneration. Finally, existing bone organoid platforms are mostly based on rigid scaffolds or printed constructs that lack geometric adaptability, limiting their ability to conform to complex, patient‐specific bone defects. These challenges collectively restrict the clinical translation of bone organoid technology and underscore the urgent need for integrated solutions that address oxygenation, immune modulation, and morphological flexibility.

**Figure 1:**
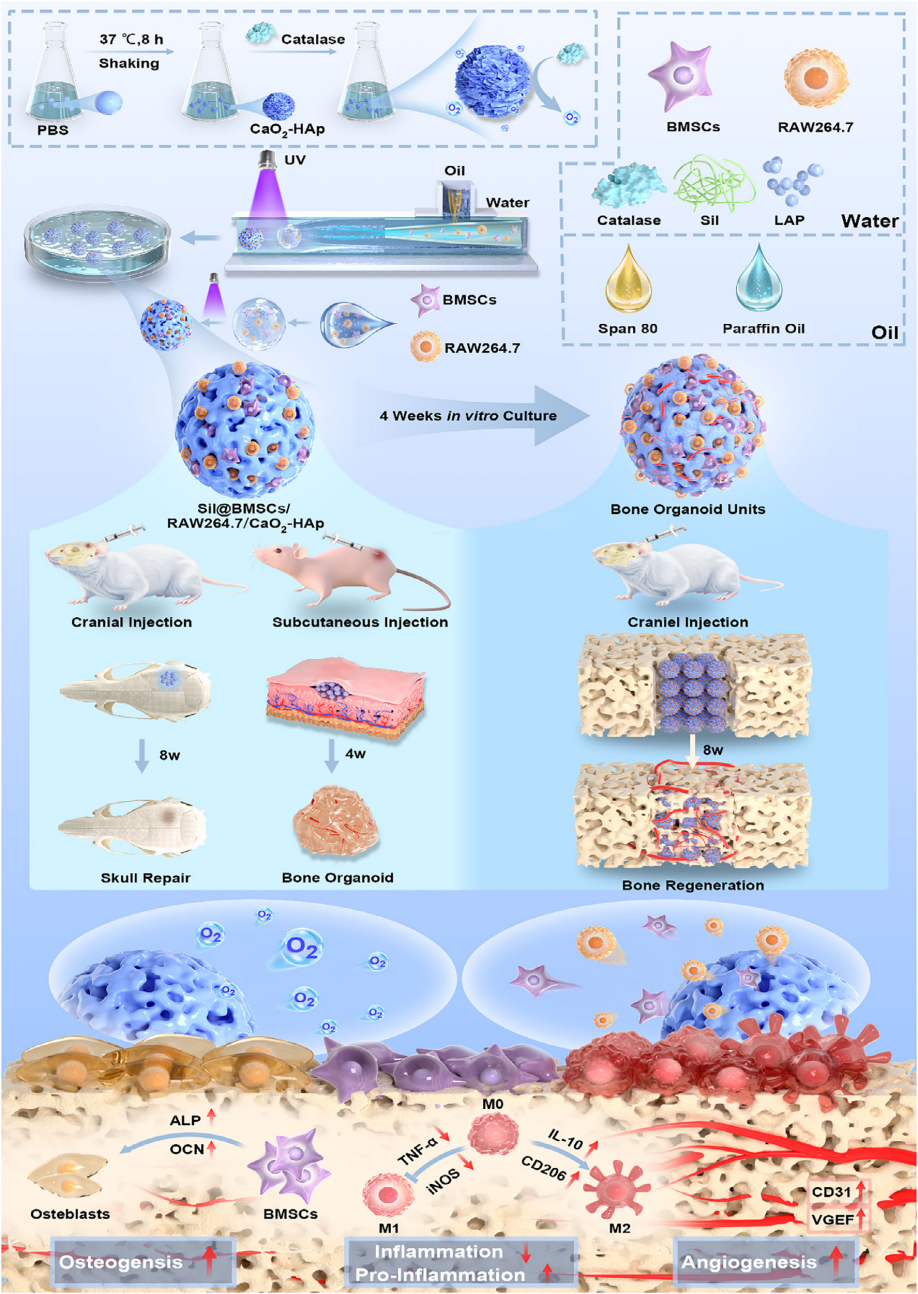
Schematic diagram of the design and application of oxygen‐releasing immunomodulatory microspheres for bone organoid construction and bone regeneration.

To address these challenges, this study proposes an innovative microfluidic hydrogel microsphere strategy to construct bone organoid units capable of sustained oxygen release, immune modulation, and conformal repair (**Figure** **1**). By utilizing microfluidic technology, silk fibroin hydrogel is employed as a supporting material to encapsulate calcium peroxide (CaO₂) particles, bone marrow mesenchymal stem cells (BMSCs), and macrophages (RAW264.7) within microspheres. This approach not only ensures continuous oxygen release, alleviating the hypoxic conditions in bone organoids but also promotes osteogenesis and controls inflammation through immune modulation. Additionally, this strategy allows precise control over the size, shape, and composition of the microspheres, enabling the creation of shape‐matched bone organoid units tailored for various bone defect sites and sizes. By filling customized bone organoid units, this system effectively mimics natural bone healing processes, promoting osteogenesis, angiogenesis, and immune modulation. In vitro experiments demonstrated significant osteogenic mineralization and maintained immune regulatory functions for up to 28 days. In vivo experiments showed that the bone organoid units using this strategy significantly accelerated new bone formation at critical‐sized cranial defects, enhanced angiogenesis, and reduced inflammation, highlighting their potential for large bone defect repair. This innovative strategy offers a personalized, customizable approach to bone defect repair, with significant clinical application potential. By precisely controlling the structure and function of bone organoids and combining the needs for oxygen supply, immune modulation, and shape‐matched repair, this approach presents a novel therapeutic pathway for bone repair, paving the way for future clinical treatments of large bone defects.

## 2 Results and Discussion

### 2.1 Preparation and Characterization of Hydrogel Microspheres and Oxygen Releasing Materials

In addressing the multiple challenges of bone organoid construction, including insufficient oxygen supply and the need for immune modulation, we observed in previous studies that bone organoids undergo rapid cell apoptosis at the center due to inadequate oxygen supply during long‐term culture.^[^ ^15^ ^]^ Moreover, immune microenvironment regulation is critical in bone repair.^[^ ^21^ ^]^ To address these issues, we developed a microfluidic hydrogel microsphere strategy capable of precisely controlling the size and shape of the microspheres and effectively encapsulating oxygen‐releasing materials to mitigate the detrimental effects of hypoxic environments on osteogenesis. This strategy also incorporates immune‐modulatory factors to further promote immune response control during bone repair.

**Figure 2:**
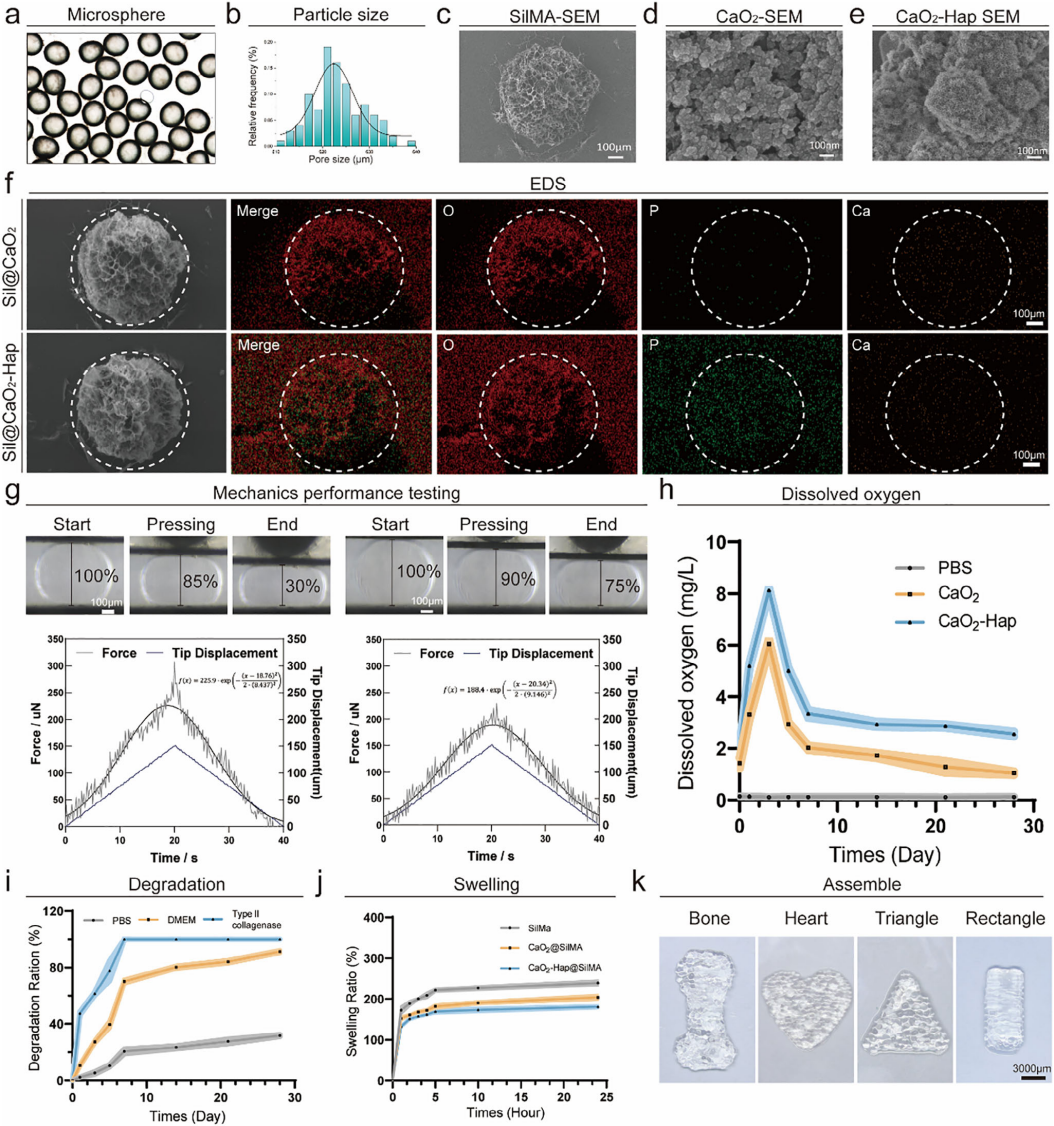
Characterization of hydrogel microspheres and oxygen‐releasing materials. a) Optical microscopy images of SilMA microspheres. b) Size distribution of SilMA microspheres. c) SEM image of SilMA microspheres showing their porous structure. d,e) SEM images of CaO₂ and CaO₂‐HAp particles, respectively. f) EDS mapping images of SilMA@CaO₂ and SilMA@CaO₂‐HAp microspheres, with calcium (Ca) and phosphorus (P) signals confirming the successful encapsulation of CaO₂‐HAp in the microspheres. g) Force‐displacement test of SilMA microspheres and SilMA@CaO₂‐HAp microspheres under compression. h) Oxygen release profiles of SilMA@CaO₂ and SilMA@CaO₂‐HAp microspheres over 30 days (*n* = 3). i) Degradation profiles of SilMA@CaO₂‐HAp microspheres tested in PBS, DMEM, and type II collagenase (*n* = 3). j) Swelling behavior of SilMA microspheres, SilMA@CaO₂, and SilMA@CaO₂‐HAp microspheres (*n* = 3). k) Conformal assembly of SilMA@CaO₂‐HAp microspheres into customizable shapes, including bone, heart, triangle, and rectangle.

The microfluidic platform was equipped with coaxial needles to produce uniform hydrogel microspheres (**Figure** **2**a). The water phase, containing silk fibroin methacryloyl and lithium phenyl (2,4,6‐trimethylbenzoyl) phosphinate (LAP) was delivered through the inner needle, while the outer needle transported the oil phase, which encapsulated the aqueous droplets through shear force.^[^ ^22^ ^]^ By carefully controlling the flow rate ratio between the aqueous and oil phases, we successfully generated microspheres with an average diameter of ≈622.6 ± 5.23 mm, as shown by particle size distribution analysis (Figure 2b). Under 365 nm UV irradiation, the SilMA in the aqueous phase underwent crosslinking with the LAP, resulting in the formation of stable, porous hydrogel microspheres. This porous structure is beneficial for the encapsulation of functional agents and the delivery of nutrients (Figure 2c).

To enable oxygen release, we synthesized CaO₂ and calcium peroxide‐hydroxyapatite (CaO₂‐HAp) particles (Figure 2d,e), which were then incorporated into the aqueous SilMA solution prior to microsphere fabrication. This approach allowed for the encapsulation of these particles within the hydrogel microspheres, providing sustained oxygen release. Energy‐dispersive X‐ray spectroscopy (EDS) confirmed the successful loading of CaO₂ and CaO₂‐HAp, as indicated by distinct calcium (Ca) and phosphorus (P) signals within the microspheres (Figure 2f). The Figure 2g demonstrates the compressive deformation and force‐displacement relationship of SilMA microspheres (left) and SilMA microspheres encapsulating CaO₂‐HAp (right). Images of the microspheres at the start, under pressing, and at the end of compression indicate their deformation percentages. SilMA Microspheres: The deformation analysis shows progressive compression from 100% at the beginning to 30% at the end. The force‐displacement graph reveals a peak force of 225.9 µN, fitting a Gaussian curve $f\left(x\right)=225.9\cdot \exp\left[-\frac{{\left(x-18.76\right)}^{2}}{2\cdot {\left(8.437\right)}^{2}}\right]$, reflecting the elastic and subsequent failure characteristics of the microspheres. SilMA microspheres encapsulating CaO₂‐HAP: These microspheres demonstrate enhanced resistance to deformation. The peak force in the force‐displacement graph is lower at 188.4 µN, with a broader Gaussian curve $f\left(x\right)=188.4\cdot \exp\left[-\frac{{\left(x-20.34\right)}^{2}}{2\cdot {\left(9.146\right)}^{2}}\right]$, suggesting improved structural integrity and resilience under compression. These results highlight the reinforcement effect of CaO₂‐HAp encapsulation, enhancing the deformation resistance of SilMA microspheres. Mechanical testing revealed that the CaO₂‐HAp‐loaded microspheres exhibited superior resilience under compression compared to CaO₂‐only microspheres, ensuring structural stability in physiological environments.

Oxygen release studies demonstrated that CaO₂‐HAp microspheres provided sustained oxygen delivery over a 30‐day period, significantly outperforming both CaO₂‐only microspheres and PBS controls (Figure 2h). This long‐term oxygen release helps alleviate the hypoxic conditions typically encountered in long‐term in vitro bone organoid cultures and large bone defects, promoting cell survival and enhancing osteogenesis. Furthermore, degradation and swelling studies showed that CaO₂‐HAp microspheres exhibited controlled degradation and moderate swelling behavior, maintaining structural integrity while offering functional adaptability (Figure 2i,j). Finally, these microspheres demonstrated excellent conformability, enabling them to be assembled into customized shapes, such as bone‐like structures or geometric forms, facilitating personalized repair strategies for irregular bone defects (Figure 2k).

This innovative oxygen‐releasing hydrogel microsphere system addresses key challenges in bone organoid engineering, including oxygen supply and conformability. It provides a multifunctional and customizable platform for bone defect repair, with the potential to accelerate bone regeneration. By integrating mechanical stability and functional oxygen release, this approach lays a solid foundation for advancing bone organoid research and clinical applications in large‐scale bone defect repair.

### 2.2 Effects of Oxygen‐Releasing SilMA Microspheres on Cell Viability, Proliferation, Osteogenesis, and Immunomodulation

Building upon the innovative strategy of constructing conformal bone organoids using oxygen‐releasing silk fibroin hydrogel microspheres, this section explores the in vitro performance of hydrogel microspheres encapsulating BMSCs, macrophages (RAW264.7), and oxygen‐releasing materials (CaO₂ and CaO₂‐HAp) in terms of cell survival, proliferation, osteogenesis, and immunomodulation. Four experimental groups were designed: Sil@B (silk fibroin hydrogel microspheres encapsulating BMSCs), Sil@B/R (microspheres encapsulating BMSCs and RAW264.7), Sil@B/R/C (microspheres encapsulating BMSCs, RAW264.7, CaO₂, and catalase), and Sil@B/R/C‐H (microspheres encapsulating BMSCs, RAW264.7, CaO₂‐HAp, and catalase). The focus was to investigate the effects of oxygen release and immunomodulation on cellular behaviors.

**Figure 3:**
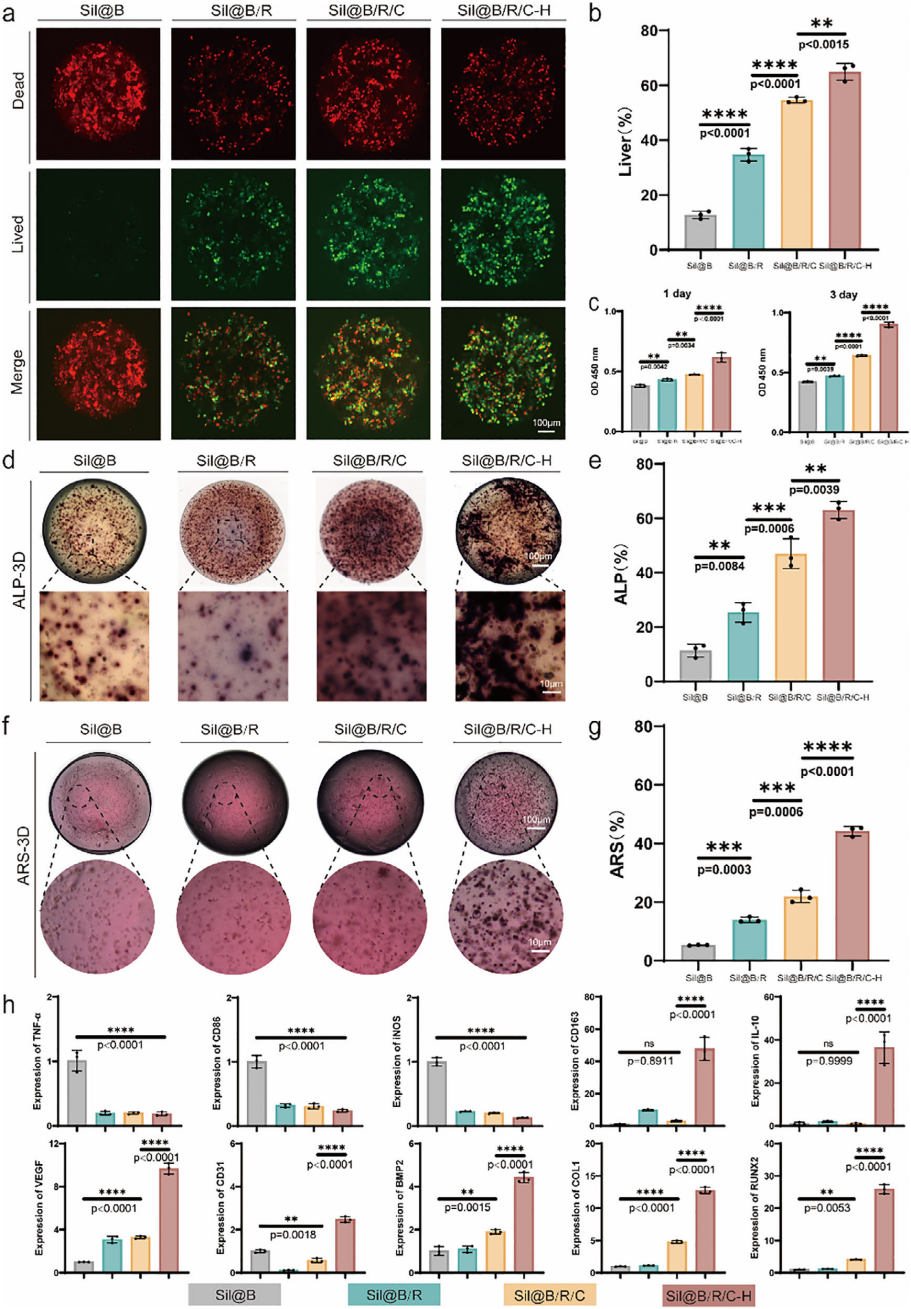
Effects of oxygen‐releasing SilMA microspheres on cell viability, proliferation, osteogenesis, and immunomodulation. a) Live/dead staining of cells in Sil@B, Sil@B/R, Sil@B/R/C, and Sil@B/R/C‐H microspheres under hypoxic conditions (1 day) and normoxic conditions (2 days). b) Quantitative analysis of cell viability based on live/dead staining. c) CCK‐8 assay showing the proliferation of cells in microspheres under hypoxic (1 day) and normoxic (2 days) conditions. d) ALP staining of microspheres showing osteogenic differentiation. e) Quantitative analysis of ALP staining. f) ARS staining showing calcium deposition of cells in microspheres during osteogenesis. g) Quantitative analysis of ARS staining. h) Gene expression analysis of inflammatory markers (TNF‐α, CD86, and iNOS), anti‐inflammatory markers (CD163 and IL‐10), angiogenic markers (VEGF, CD31), and osteogenic markers (BMP‐2, COL1, and RUNX2) in cells encapsulated within microspheres. Data are presented as means ± SD (*n* = 3). ^*^ *p* < 0.05, ^**^ *p* < 0.01, ^***^ *p* < 0.001, ^****^ *p* < 0.0001.

Cell survival and proliferation of BMSCs within oxygen‐releasing silk fibroin hydrogel microspheres were assessed under hypoxic (1 day) and normoxic (2 days) conditions using live/dead staining and CCK‐8 assays. Live/dead staining results and quantitative analysis (**Figure** **3**a,b) showed that Sil@B/R/C‐H microspheres significantly improved cell survival compared to other groups, as evidenced by a higher proportion of live cells (green fluorescence) and a marked reduction in dead cells (red fluorescence). This indicates that the oxygen‐releasing material CaO₂‐HAp effectively mitigates hypoxic conditions within the microspheres, providing a more favorable microenvironment for cell survival. The CCK‐8 assay further confirmed these findings. Under hypoxic conditions, Sil@B/R/C and Sil@B/R/C‐H microspheres demonstrated significantly higher cell viability compared to Sil@B and Sil@B/R groups (Figure 3c), highlighting the critical role of oxygen regulation. After two days of culture under normoxic conditions, Sil@B/R/C‐H microspheres exhibited the most pronounced cell proliferation activity (Figure 3c), suggesting a synergistic effect between sustained oxygen release and bioactive components.

Osteogenic differentiation of BMSCs within the microspheres was evaluated through Alkaline Phosphatase (ALP) and Alizarin Red S (ARS) staining, which represent early and late osteogenic markers, respectively. As shown in Figure 3d,e, ALP activity was significantly enhanced in the Sil@B/R/C and Sil@B/R/C‐H groups, with Sil@B/R/C‐H microspheres exhibiting the highest osteogenic potential. ARS staining further revealed a substantial increase in mineralized matrix formation in the Sil@B/R/C‐H group (Figure 3f). Quantitative analysis (Figure 3g) indicated that ARS activity in Sil@B/R/C‐H microspheres was significantly higher than in other groups, underscoring the pivotal role of sustained oxygen release in promoting osteogenic mineralization.

To investigate the immunomodulatory effects of oxygen‐releasing microspheres, gene expression analysis was performed to assess key markers related to inflammation, angiogenesis, and osteogenesis (Figure 3h; Figure S1, Supporting Information). The Sil@B/R/C‐H group significantly downregulated pro‐inflammatory markers such as TNF‐α, iNOS, CD86, IL‐1β, and IL‐6 while upregulating anti‐inflammatory markers such as IL‐10, CD163, TGF‐β and Arg‐1,^[^ ^23^ ^]^ indicating effective regulation of immune responses and macrophage polarization toward the M2 phenotype. Additionally, the Sil@B/R/C‐H group significantly upregulated markers associated with angiogenesis such as VEGF and CD31, and osteogenesis such as BMP‐2, RUNX2, and COL1 demonstrating that these oxygen‐releasing microspheres create a regenerative microenvironment conducive to bone repair. By coordinating immunomodulation with angiogenesis and osteogenesis, Sil@B/R/C‐H microspheres exhibit multifunctional potential to meet the complex demands of bone defect repair, including oxygen supply, enhanced osteogenic differentiation, reduced immune responses, and promotion of angiogenesis.

These findings demonstrate that Sil@B/R/C‐H microspheres exhibit superior performance in supporting cell survival, promoting osteogenic differentiation, and regulating immune responses through sustained oxygen release. The integration of CaO₂‐HAp not only effectively alleviates hypoxic conditions but also establishes a regenerative microenvironment favorable for bone organoid development. Furthermore, by encapsulating RAW264.7 immune cells, this strategy offers a novel approach to bone regeneration and immunomodulation. It provides a solid theoretical and practical foundation for regenerative medicine, particularly in applications requiring oxygen supply, osteogenesis, angiogenesis, and immune regulation, such as the construction of bone organoids and the repair of critical‐sized bone defects.

### 2.3 Transcriptomic Analysis of Oxygen‐Releasing Immunomodulatory Microspheres

To investigate the molecular mechanisms underlying the enhanced osteogenesis, angiogenesis, and immunomodulation induced by oxygen‐releasing immunomodulatory microspheres, we performed transcriptomic sequencing on four experimental groups: Sil@B, Sil@B/R, Sil@B/R/C, and Sil@B/R/C‐H. Each group included three parallel biological replicates to ensure the reliability of the results. This comprehensive transcriptomic analysis revealed key differences in gene expression and enriched pathways, providing mechanistic insights into the synergistic effects of immune regulation and sustained oxygen release in promoting bone regeneration and vascularization.

**Figure 4:**
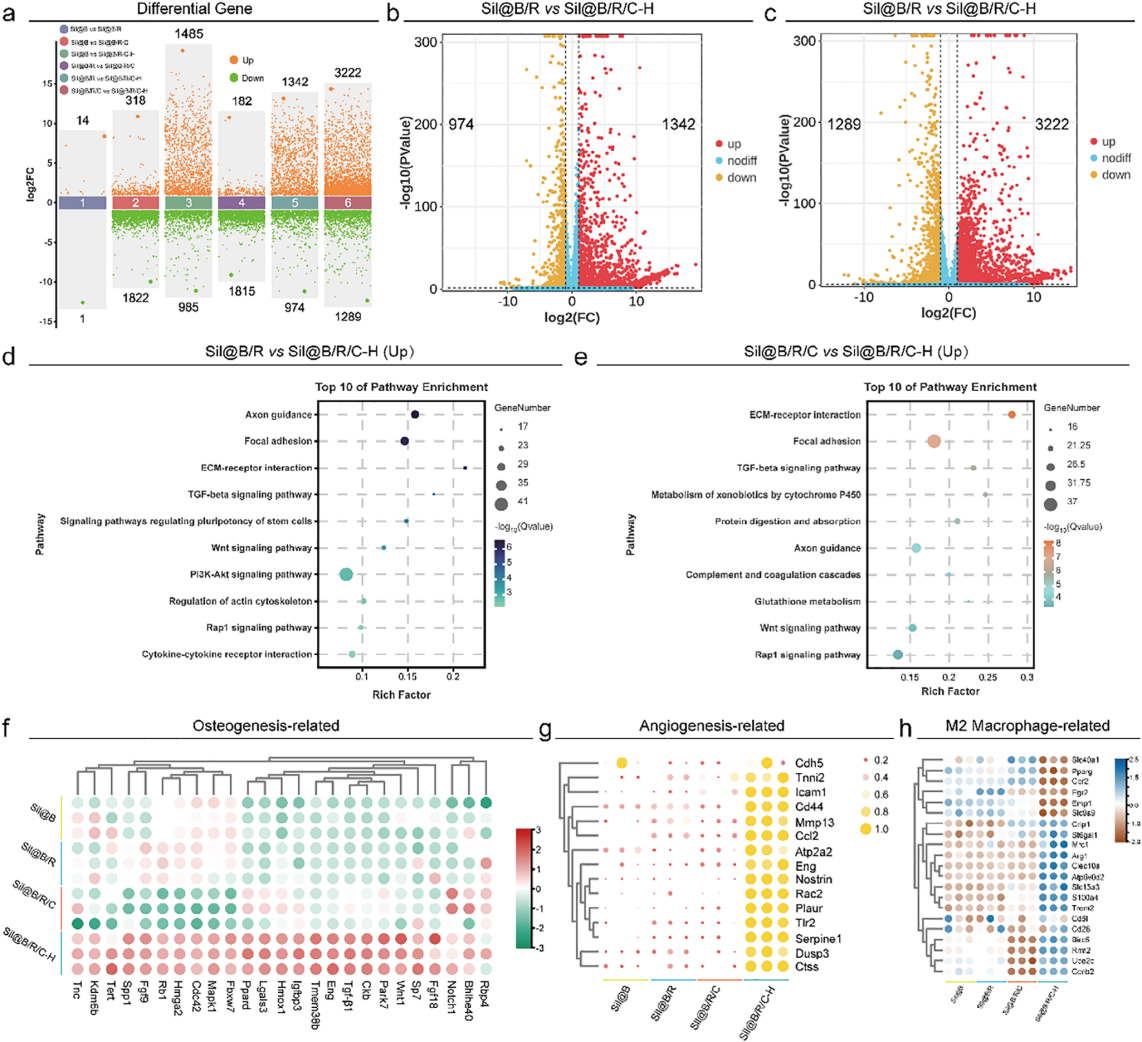
Transcriptomic analysis of oxygen‐releasing immunomodulatory microspheres. a) DEGs among four groups (Sil@B, Sil@B/R, Sil@B/R/C, and Sil@B/R/C‐H). Volcano plots showing upregulated and downregulated genes in the Sil@B/R versus Sil@B/R/C‐H b) and Sil@B/R/C versus Sil@B/R/C‐H c). Pathway enrichment analysis of upregulated DEGs in the Sil@B/R versus Sil@B/R/C‐H d) and Sil@B/R/C versus Sil@B/R/C‐H e). f) Heatmap of osteogenesis‐related genes across all groups. g) Heatmap of angiogenesis‐related genes across all groups. h) Heatmap of M2 macrophage polarization‐related genes across all groups. Data are presented as means ± SD (*n* = 3). ^*^ *p* < 0.05, ^**^ *p* < 0.01, ^***^ *p* < 0.001, ^****^ *p* < 0.0001.

Differential gene expression (DGE) analysis highlighted significant variations between treatment groups (**Figure** **4**a). Among the comparisons, notable differences were observed between Sil@B/R and Sil@B/R/C‐H, as well as between Sil@B/R/C and Sil@B/R/C‐H, with the latter group exhibiting a substantially higher number of differentially expressed genes (DEGs). Other group comparisons, such as Sil@B versus Sil@B/R and Sil@B versus Sil@B/R/C, showed minimal differences, suggesting that the enhanced osteogenesis and vascularization are primarily attributed to the Sil@B/R/C‐H microspheres.

Volcano plots (**Figure** **4**b,c) further demonstrated the significance of DEGs in Sil@B/R versus Sil@B/R/C‐H and Sil@B/R/C versus Sil@B/R/C‐H comparisons. The Sil@B/R/C‐H group showed a higher number of upregulated genes compared to the other groups, indicating its superior ability to activate genes associated with bone regeneration, angiogenesis, and immune modulation.

Pathway enrichment analysis of upregulated DEGs identified several critical signaling pathways involved in bone regeneration and vascularization. In the Sil@B/R versus Sil@B/R/C‐H comparison (Figure 4d), top enriched pathways included focal adhesion, ECM‐receptor interaction, TGF‐β signaling, Wnt signaling, and PI3K‐Akt signaling. These pathways are well‐documented for their roles in osteogenesis, angiogenesis, and extracellular matrix (ECM) remodeling. Similarly, the Sil@B/R/C versus Sil@B/R/C‐H comparison (Figure 4e) revealed similar enrichment trends, with additional pathways such as Rap1 signaling and axon guidance also being activated, further emphasizing the regulatory role of Sil@B/R/C‐H microspheres in promoting tissue regeneration.

The osteogenic potential of the microspheres was confirmed by the expression profiles of key osteogenesis‐related genes (Figure 4f). The Sil@B/R/C‐H group demonstrated significantly higher expression of pivotal osteogenic markers such as Tgf‐β1, Notch1, and SP7 compared to other groups.^[^ ^24^ ^]^ These genes play essential roles in osteogenic differentiation, bone matrix formation, and mineralization. The upregulation of these markers strongly supports the enhanced osteogenic capacity of Sil@B/R/C‐H microspheres.

Effective vascularization is essential for successful bone regeneration.^[^ ^25^ ^]^ Gene expression analysis revealed significantly higher levels of angiogenesis‐related markers such as Icam1 in the Sil@B/R/C‐H group (Figure 4g). These genes are critical for endothelial cell proliferation, migration, and new blood vessel formation.^[^ ^26^ ^]^ The upregulation of these markers in the Sil@B/R/C‐H group indicates its superior angiogenic potential, which likely contributes to improved nutrient and oxygen delivery to the regenerating bone tissue.

Immune regulation is a critical factor in bone regeneration. The expression of M2 macrophage polarization‐related genes was significantly upregulated in the Sil@B/R/C‐H group (Figure 4h). Notably, Arg1 and other pro‐healing M2 macrophage markers showed the highest expression levels in this group, suggesting that the Sil@B/R/C‐H microspheres effectively promoted an anti‐inflammatory and pro‐regenerative immune environment.^[^ ^27^ ^]^ This finding is consistent with the immunomodulatory effects observed in earlier sections, where the Sil@B/R/C‐H group demonstrated enhanced macrophage polarization and reduced inflammation at the bone defect site.

This transcriptomic analysis provides valuable insights into the molecular mechanisms underlying the enhanced osteogenic, angiogenic, and immunomodulatory effects of oxygen‐releasing immunomodulatory microspheres. The Sil@B/R/C‐H group demonstrated significant upregulation of genes involved in bone formation, vascularization, and M2 macrophage polarization, suggesting a synergistic interaction between sustained oxygen release and immune regulation. Key signaling pathways such as TGF‐β, Wnt, and ECM‐receptor interaction were identified as central mediators of these effects, further validating the role of Sil@B/R/C‐H microspheres in creating a regenerative microenvironment.

Overall, these findings reinforce the hypothesis that the integration of oxygen release and immune modulation within microspheres can effectively address the challenges of hypoxia and inflammation in bone regeneration. By promoting osteogenesis, angiogenesis, and tissue repair, the Sil@B/R/C‐H microspheres lay a solid foundation for the construction of functional bone organoid units and offer a promising platform for advanced bone tissue engineering and regenerative medicine applications.

### 2.4 Evaluation of Oxygen‐Releasing Immunomodulatory Microspheres for In Vivo Bone Regeneration

Based on the in vitro results and material design principles, this section presents the in vivo evaluation of silk fibroin oxygen‐releasing immunomodulatory microspheres in bone regeneration. Using a mouse critical‐sized calvarial defect model, the ability of microspheres encapsulating BMSCs, RAW264.7 macrophages, and oxygen‐releasing materials to promote osteogenesis and immunomodulation was assessed. Experimental groups (Sil@B, Sil@B/R, Sil@B/R/C, and Sil@B/R/C‐H) were compared with an untreated control group (Con) at 4 and 8 weeks. Micro‐CT imaging, histological staining, immunohistochemistry, and immunofluorescence analyses were used to reveal the synergistic effects of sustained oxygen supply and immunomodulation in bone repair.

**Figure 5:**
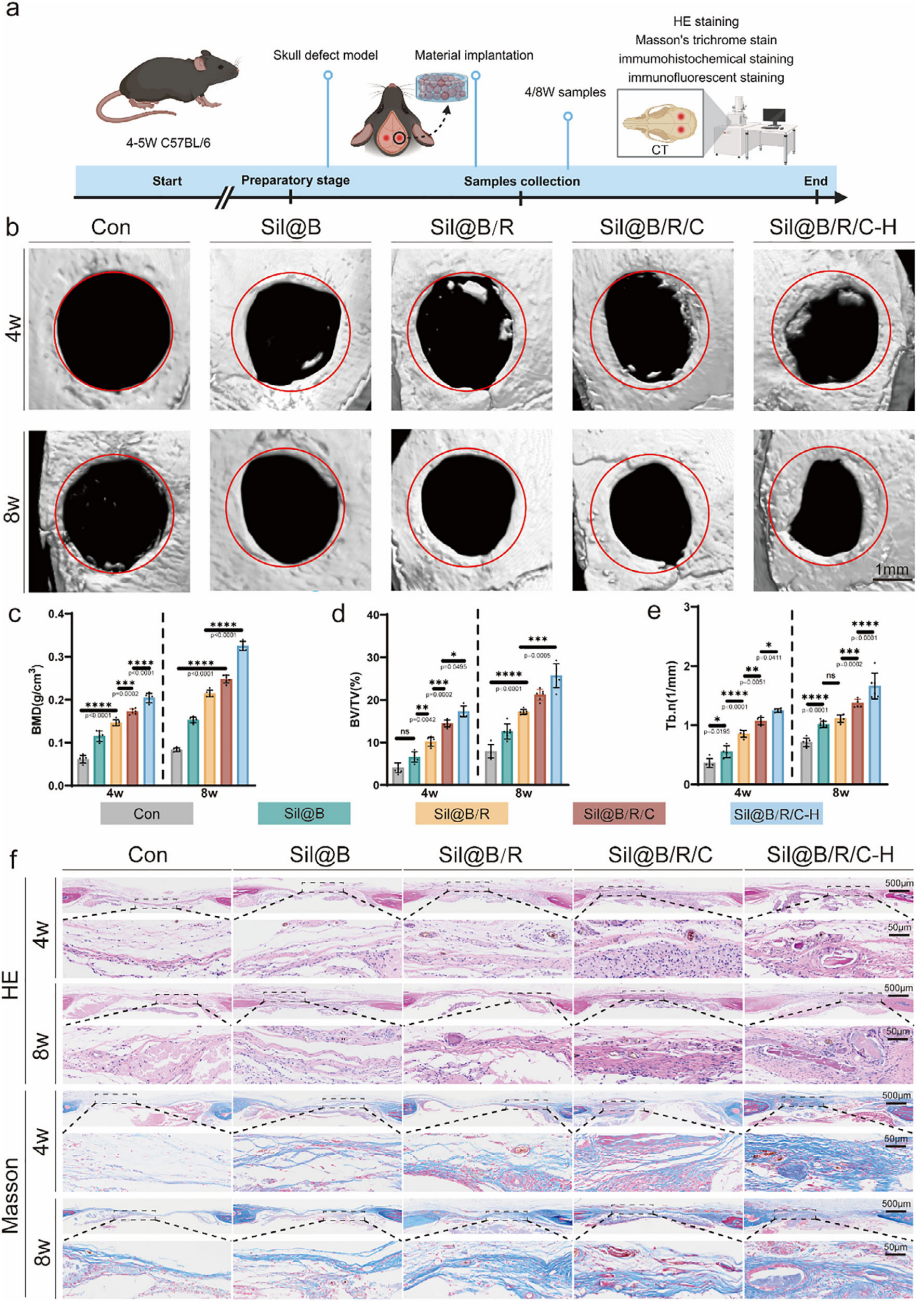
In vivo evaluation of oxygen‐releasing SilMA microspheres in a mouse calvarial critical‐sized defect model. a) Schematic illustration of the experimental design for bone regeneration evaluation, including the skull defect model, material implantation, and analysis at 4 and 8 weeks. b) Micro‐CT images of bone regeneration in control (Con), Sil@B, Sil@B/R, Sil@B/R/C, and Sil@B/R/C‐H groups at 4 and 8 weeks. c–e) Quantitative analysis of BMD, BV/TV, and Tb. N in the defect areas. f) H&E and Masson's trichrome staining of tissue sections from the defect areas at 4 and 8 weeks, showing bone regeneration and collagen deposition. Data are presented as means ± SD (*n* = 6). ^*^ *p* < 0.05, ^**^ *p* < 0.01, ^***^ *p* < 0.001, ^****^ *p* < 0.0001.

Micro‐CT imaging demonstrated clear differences in bone regeneration among the experimental groups (**Figure** **5**a,b). Consistent with the in vitro findings, the control group exhibited minimal new bone formation at both 4 and 8 weeks. Similarly, the Sil@B group, lacking immune cells and oxygen‐releasing components, showed limited bone repair, indicating that insufficient bioactivity and oxygen supply hindered bone regeneration. In contrast, the Sil@B/R/C‐H group displayed the most significant bone regeneration, with substantial bone deposition observed as early as 4 weeks and dense, mature bone structures by 8 weeks. The other experimental groups (Sil@B/R and Sil@B/R/C) exhibited moderate levels of bone repair, highlighting the critical role of sustained oxygen release and macrophage‐mediated immunomodulation in bone regeneration. These findings directly support the hypothesis that.^[^ ^28^ ^]^ Quantitative analyses of bone mineral density (BMD), bone volume‐to‐total volume ratio (BV/TV), and trabecular number (Tb.N) further confirmed these observations (**Figure** **5**c–e). The Sil@B/R/C‐H group showed significantly higher BMD, BV/TV, and Tb.N values at both 4 and 8 weeks compared to the other groups, demonstrating the crucial role of CaO₂‐HAp in overcoming hypoxia and supporting immunomodulatory functions. These results suggest that the inclusion of CaO₂‐HAp in the microspheres ensures adequate oxygen supply, effectively addressing the typical hypoxic environment in critical‐sized defects. Additionally, the immunomodulatory effects of RAW264.7 macrophages may regulate inflammation and promote angiogenesis, thereby creating a favorable environment for osteogenesis and fundamentally improving bone regeneration outcomes.

Histological staining provided supplementary evidence for the bone regeneration process. H&E staining (Figure 5f) showed disorganized tissue and persistent defects in the control and Sil@B groups at both 4 and 8 weeks. The Sil@B/R group exhibited some bone formation, but the tissue structure remained incomplete. The Sil@B/R/C group showed improved tissue structure, though with limited bone maturity. However, the Sil@B/R/C‐H group displayed organized and vascularized bone structures at 4 weeks, with mature bone tissue and reduced fibrotic tissue observed at 8 weeks. These findings underscore the importance of oxygen supply and immunomodulation in accelerating matrix remodeling and bone maturation. Masson's trichrome staining further emphasized these trends, with the Sil@B/R/C‐H group exhibiting more extensive collagen deposition and mineralization compared to the control and Sil@B groups. These results strongly support the hypothesis that the incorporation of macrophages and oxygen‐releasing materials into the microspheres promotes ECM remodeling and effective bone repair.^[^ ^29^ ^]^

**Figure 6:**
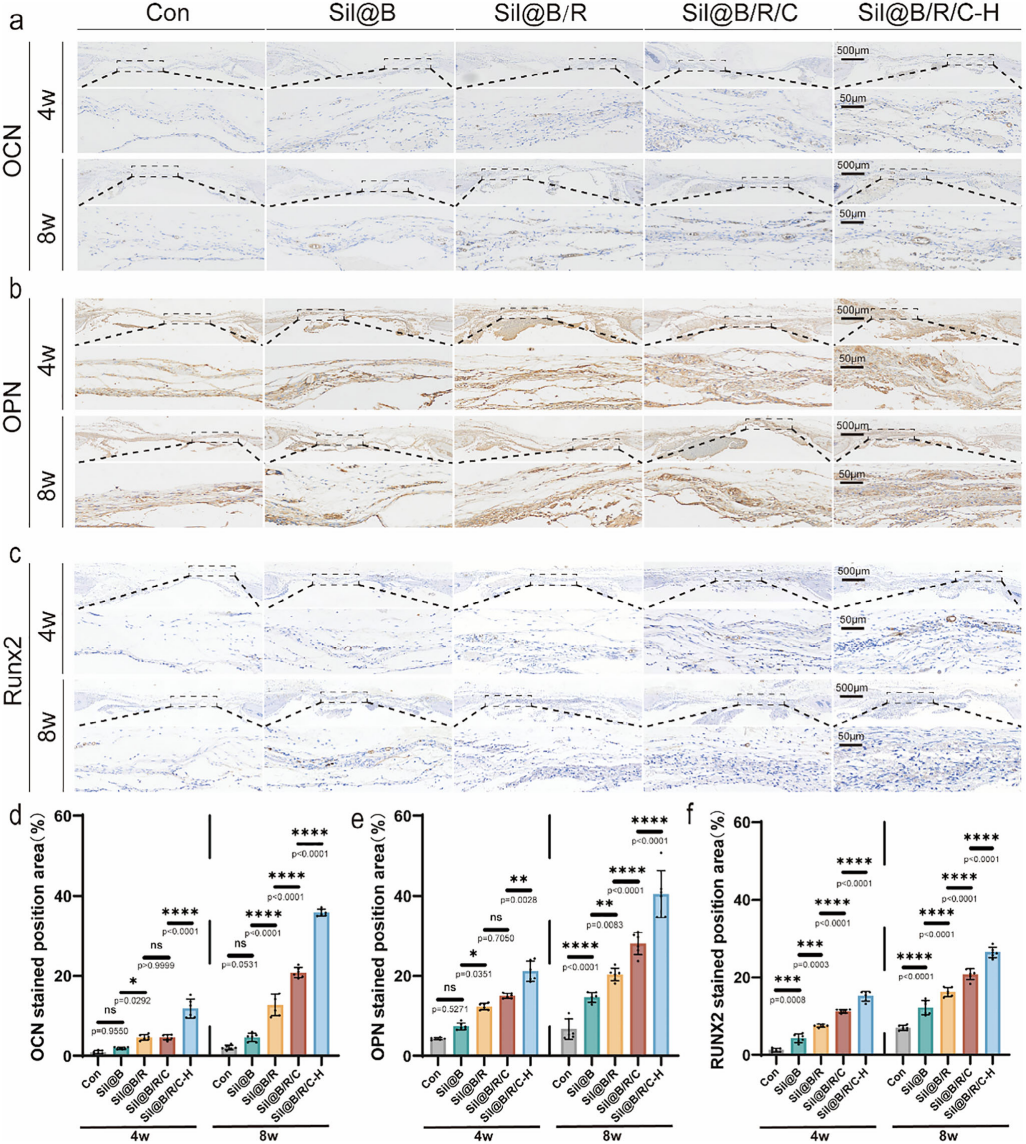
Immunohistochemical analysis of osteogenic markers in defect areas treated with oxygen‐releasing SilMA microspheres. a–c) Immunohistochemical staining for OCN, OPN, and RUNX2 in the defect areas at 4 and 8 weeks in control (Con), Sil@B, Sil@B/R, Sil@B/R/C, and Sil@B/R/C‐H groups. d–f) Quantitative analysis of the stained areas for OCN, OPN, and RUNX2. Data are presented as means ± SD (*n* = 6). ^*^ *p* < 0.05, ^**^ *p* < 0.01, ^***^ *p* < 0.001, ^****^ *p* < 0.0001.

To evaluate in vivo osteogenic differentiation, immunohistochemical staining for key osteogenic markers‐osteocalcin (OCN), osteopontin (OPN), and RUNX2‐was performed (**Figure** **6**a,c). Consistent with the in vitro data, the expression of OCN and OPN was significantly higher in the Sil@B/R/C and Sil@B/R/C‐H groups compared to the control and Sil@B groups. RUNX2, as a marker of osteogenic activity, was elevated in the Sil@B/R/C and Sil@B/R/C‐H groups at 4 weeks and further increased at 8 weeks, indicating sustained promotion of osteogenesis. Quantitative analysis of the stained areas (Figure 6d,f) showed the highest marker expression in the Sil@B/R/C‐H group at both time points, especially at 8 weeks. These findings align with the previous conclusions that sustained oxygen release improves the hypoxic environment, supporting BMSC activity and promoting osteogenic differentiation.

**Figure 7:**
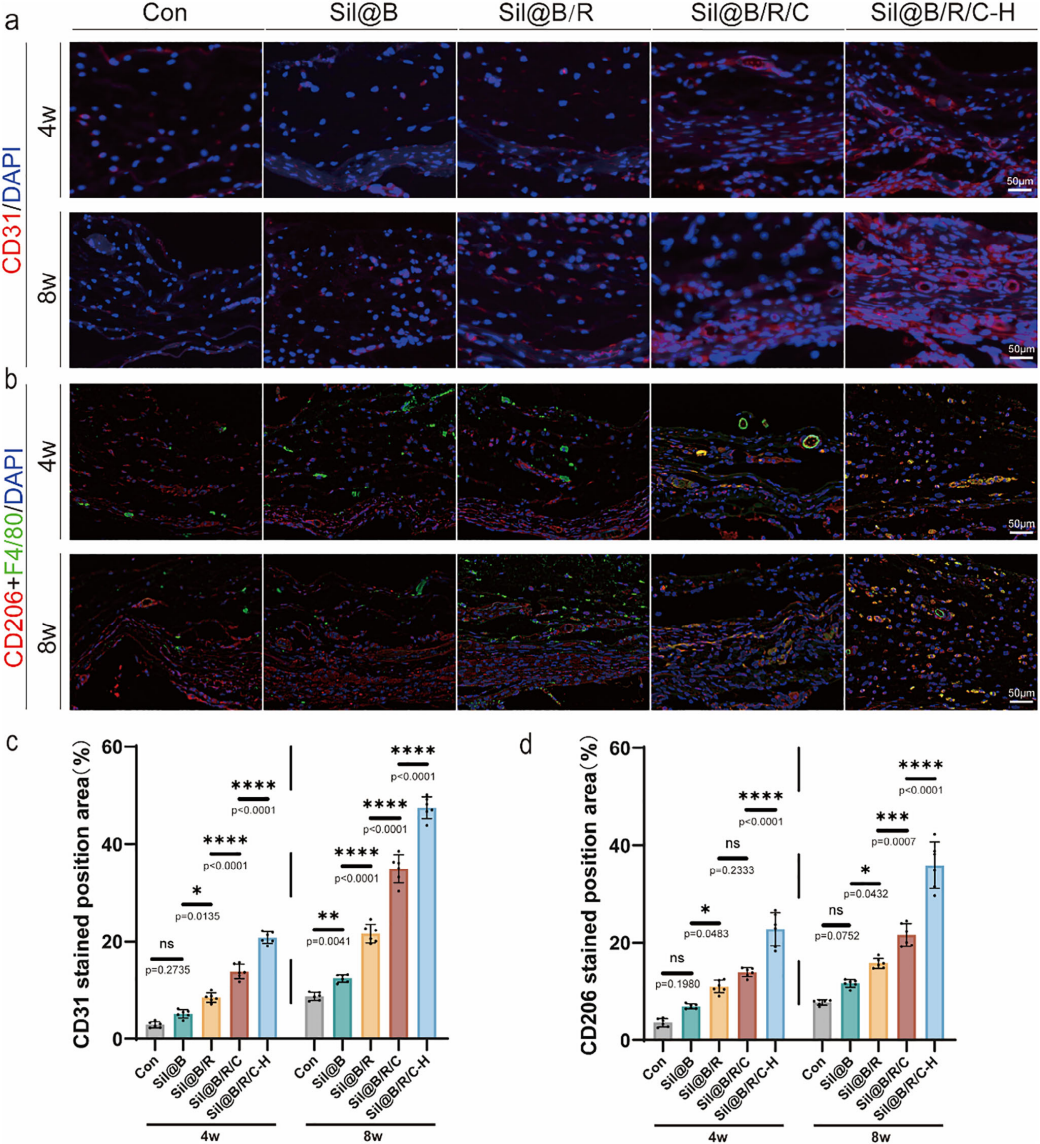
Immunofluorescence analysis of angiogenic and immunomodulatory markers in defect areas treated with oxygen‐releasing SilMA microspheres. a) Immunofluorescence staining for CD31 in control (Con), Sil@B, Sil@B/R, Sil@B/R/C, and Sil@B/R/C‐H groups at 4 and 8 weeks. b) Immunofluorescence staining for CD206 in the defect areas. c, d) Quantitative analysis of CD31 and CD206 staining. Data are presented as means ± SD (*n* = 6). ^*^ *p* < 0.05, ^**^ *p* < 0.01, ^***^ *p* < 0.001, ^****^ *p* < 0.0001.

Immunofluorescence staining for CD31 and CD206 revealed the role of the microspheres in promoting vascularization and immunomodulation (**Figure** **7**a,b). The Sil@B/R/C‐H group exhibited the strongest angiogenic response, with significantly higher CD31‐positive staining compared to the other groups (Figure 7c). This result aligns with the emphasis in the introduction on the critical role of angiogenesis in bone regeneration, as vascularization is essential for nutrient delivery and tissue remodeling. Simultaneously, the Sil@B/R/C‐H group showed elevated CD206 expression, indicating macrophage polarization toward the anti‐inflammatory M2 phenotype. These results demonstrate that the inclusion of RAW264.7 macrophages and sustained oxygen release synergistically promoted the formation of an anti‐inflammatory environment, consistent with the immunomodulatory effects discussed in Section 2.2. Notably, the Sil@B/R/C‐H microspheres effectively regulated inflammation while promoting angiogenesis, underscoring their multifunctional potential in bone regeneration. To evaluate the systemic biocompatibility and potential toxicity of the implanted oxygen‐releasing immunomodulatory microspheres, H&E staining of major organs, including the heart, liver, spleen, lung, and kidney, was performed. No noticeable histopathological abnormalities or inflammatory infiltration were observed across all groups, indicating that the microspheres possess favorable systemic biocompatibility (Figure S2, Supporting Information).

The in vivo results validate the previous design and in vitro findings. Combining macrophages and CaO₂‐HAp within silk fibroin hydrogel microspheres effectively addressed the dual challenges of hypoxia and inflammation, which are key limitations in bone regeneration. The synergistic effects of immunomodulation and oxygen supply observed in the Sil@B/R/C‐H group not only satisfied the conformability requirements for repairing defects of different sizes and shapes but also enhanced bone repair outcomes. These results reinforce the hypothesis that immune regulation and sustained oxygen supply are essential for achieving robust and functional bone regeneration.^[^ ^30^ ^]^ By creating a regenerative microenvironment that supports osteogenesis, angiogenesis, and immunomodulation, oxygen‐releasing immunomodulatory microspheres provide a promising solution for bone organoid construction and the repair of critical‐sized bone defects.

### 2.5 Application of Oxygen‐Releasing Immunomodulatory Microspheres in Subcutaneous Bone Organoid Formation

**Figure 8:**
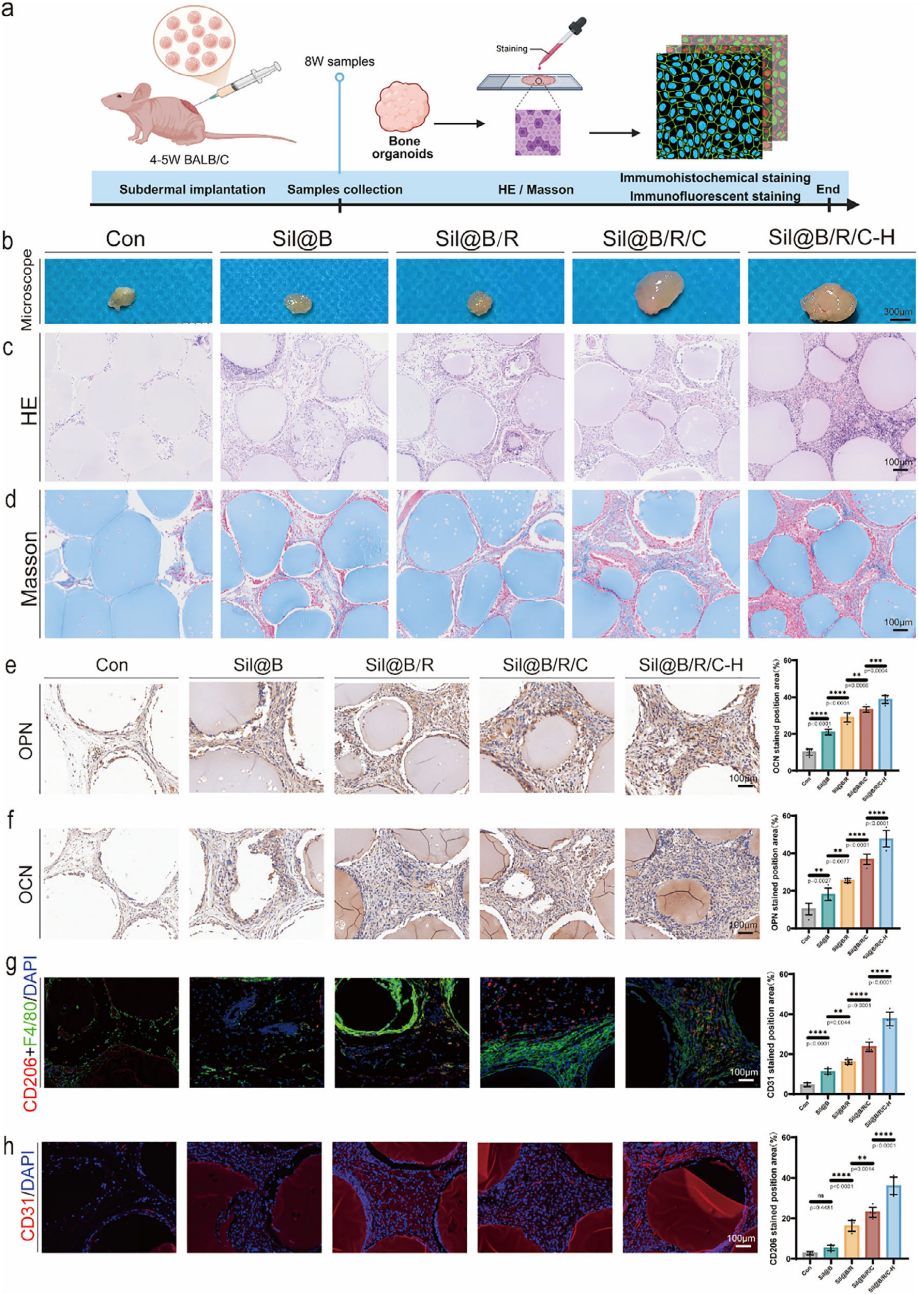
Application of oxygen‐releasing immunomodulatory microspheres in subcutaneous bone organoid formation. a) Schematic illustration of the experimental setup, including subcutaneous implantation of microspheres in BALB/c mice, sample collection at 8 weeks, and subsequent staining (HE/Masson) and immunohistochemical/immunofluorescence analysis. b) Morphological comparison of bone organoids formed in control (Con), Sil@B, Sil@B/R, Sil@B/R/C, and Sil@B/R/C‐H groups. H&E staining c) and Masson's trichrome staining d) of bone organoids. Immunohistochemical staining for OPN e) and OCN f) in bone organoids, with quantitative analysis of the stained areas indicating osteogenic activity in the experimental groups. Immunofluorescence staining for CD206, g) and CD31, h) in bone organoids, with quantitative analysis of CD206‐ and CD31‐positive areas. Data are presented as means ± SD (*n* = 6). ^*^ *p* < 0.05, ^**^ *p* < 0.01, ^***^ *p* < 0.001, ^****^ *p* < 0.0001.

To further investigate the application of oxygen‐releasing immunomodulatory microspheres in bone tissue engineering, this section evaluates their ability to support in vivo bone organoid formation. Microspheres encapsulating BMSCs, RAW264.7 macrophages, and oxygen‐releasing materials were subcutaneously implanted into BALB/c mice, resulting in the formation of bone organoids. The morphology, histology, and molecular characteristics of the bone organoids were analyzed to assess their osteogenesis, angiogenesis, and immunomodulation capabilities, providing further evidence of their regenerative potential in calvarial defect repair (**Figure** **8**a).

After 8 weeks of subcutaneous implantation, the bone organoids formed in the Sil@B/R/C‐H group were significantly larger and better structured than those in other experimental groups (Figure 8b). The control group (Con) and Sil@B group exhibited poor organoid development with no distinct tissue formation. In contrast, the Sil@B/R and Sil@B/R/C groups showed moderate growth with early signs of tissue organization. The superior size and structural integrity of the bone organoids in the Sil@B/R/C‐H group underscore the importance of the synergistic effects of sustained oxygen release and immunomodulation in promoting tissue development.

Histological staining provided detailed insights into the tissue structure and ECM remodeling within the bone organoids. H&E staining (Figure 8c) revealed well‐organized tissue structures and high cellular activity in the Sil@B/R/C‐H group, while the control and Sil@B groups exhibited sparse and disordered tissue. The Sil@B/R and Sil@B/R/C groups displayed intermediate levels of tissue formation, further emphasizing the critical roles of immune cells and sustained oxygen supply in achieving optimal tissue development. Masson's trichrome staining (Figure 8d) demonstrated extensive collagen deposition in the Sil@B/R/C‐H group, indicating significant progress in ECM remodeling and tissue maturation. In contrast, the control and Sil@B groups exhibited limited collagen deposition, reflecting their restricted regenerative capacity. These findings are consistent with the results from calvarial defect repair, further validating the importance of oxygen‐releasing immunomodulatory microspheres in promoting ECM remodeling and tissue construction.

To assess the osteogenic potential of the bone organoids, immunohistochemical staining for osteogenic markers, including OPN and OCN, was performed (Figure 8e,f). The expression of OPN and OCN was significantly higher in the Sil@B/R/C‐H group compared to other groups (*p* < 0.0001), indicating strong osteogenic activity. The Sil@B/R and Sil@B/R/C groups also showed increased expression of these markers compared to the control and Sil@B groups, albeit to a lesser extent. Quantitative analysis confirmed the highest levels of OPN and OCN expression in the Sil@B/R/C‐H group, supporting the hypothesis that sustained oxygen release and immunomodulation create a microenvironment conducive to osteogenic differentiation. These findings align with results from calvarial defect repair and in vitro studies, further demonstrating the ability of the microspheres to drive osteogenesis across different models.

To evaluate the angiogenic and immunomodulatory effects of the microspheres, immunofluorescence staining for CD31 and CD206 was conducted (Figure 8g,h). The Sil@B/R/C‐H group exhibited the strongest CD31‐positive staining, indicating significantly enhanced vascularization within the bone organoids. Similarly, CD206 expression was markedly upregulated in the Sil@B/R/C‐H group, reflecting effective polarization of macrophages toward the anti‐inflammatory M2 phenotype. The control and Sil@B groups demonstrated weak angiogenesis and immunomodulation, highlighting their limited regenerative potential under these experimental conditions. Quantitative analysis showed significantly larger CD31‐ and CD206‐positive areas in the Sil@B/R/C‐H group compared to other groups. These results further reinforce the conclusions, demonstrating that immunomodulation and angiogenesis are critical drivers of successful tissue regeneration and validating the efficacy of oxygen‐releasing immunomodulatory microspheres in promoting these processes.

The results from the subcutaneous bone organoid model further confirm the multifunctionality of Sil@B/R/C‐H microspheres in supporting bone tissue engineering. By effectively combining sustained oxygen supply with immunomodulation, these microspheres create a regenerative microenvironment conducive to osteogenesis, angiogenesis, and ECM remodeling. The outstanding performance of the Sil@B/R/C‐H group in osteogenesis, vascularization, and anti‐inflammatory effects highlights the critical role of coordinated oxygen release and immunomodulation in achieving robust and functional tissue formation.

These findings are consistent with the conclusions from earlier sections, demonstrating the translational potential of oxygen‐releasing immunomodulatory microspheres in advanced bone tissue engineering applications. Beyond repairing critical‐sized defects, this study indicates that these immunomodulatory oxygen‐releasing microspheres can be used to construct pre‐vascularized bone organoids, laying a foundation for future therapeutic strategies in complex bone defect repair.

### 2.6 Long‐Term In Vitro Culture and Osteogenic Potential of Bone Organoid Units

Building on the findings from in vitro cell experiments, in vivo cranial defect repair, and subcutaneous bone organoid formation, this section explores the potential of oxygen‐releasing immunomodulatory microspheres in generating bone organoid units through long‐term in vitro culture. A comparative study of the Sil@B group and the Sil@B/R/C‐H group was conducted to evaluate the effects of oxygen‐releasing immunomodulatory microspheres on cell viability and osteogenic performance during extended culture. The results demonstrated that Sil@B/R/C‐H microspheres could maintain high cell viability and osteogenic capacity throughout a 28‐day culture period, successfully forming functional bone organoid units. This establishes a solid foundation for the development of transplantable bone regeneration units for therapeutic applications.

**Figure 9:**
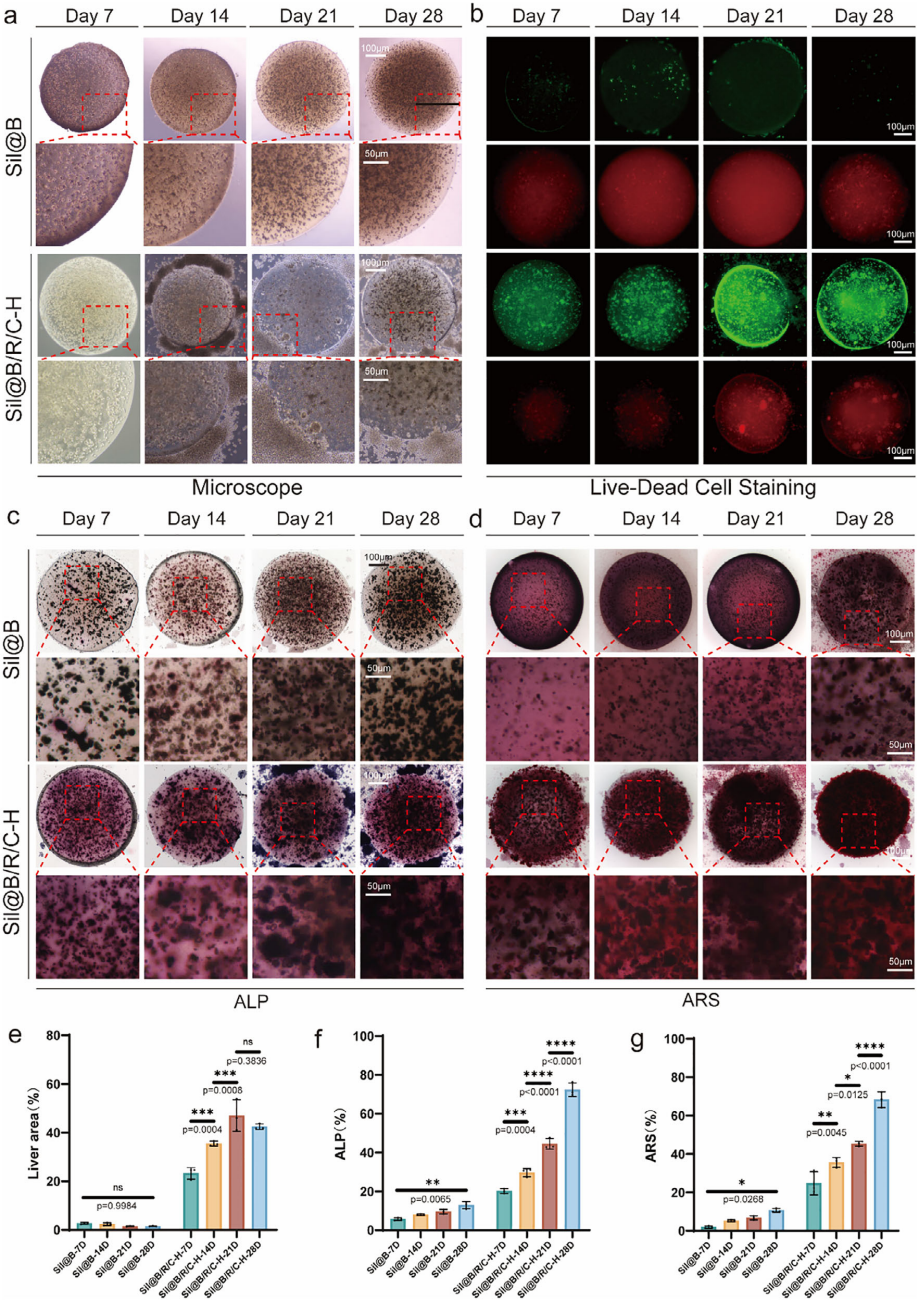
Long‐term in vitro culture and osteogenic potential of bone organoid units. a) Microscopic images of Sil@B and Sil@B/R/C‐H bone organoid units during long‐term in vitro culture (days 7, 14, 21, and 28). b) Live/dead staining of bone organoid units at different time points. c) ALP staining of bone organoid units showing osteogenic differentiation. d) ARS staining showing calcium deposition of bone organoid units during osteogenesis. Quantitative analysis of live cell area e), ALP activity f), and ARS staining g) at various time points. Data are presented as means ± SD (*n* = 3). ^*^ *p* < 0.05, ^**^ *p* < 0.01, ^***^ *p* < 0.001, ^****^ *p* < 0.0001.

Microscopic observations revealed significant differences between the experimental groups during the long‐term culture period (**Figure** **9**a). The Sil@B group exhibited progressive contraction and sparse cellular structure, indicating limited cell viability and tissue formation capacity. In contrast, the Sil@B/R/C‐H group maintained structural integrity throughout the culture period, showing pronounced cell proliferation and tissue thickening. By day 28, Sil@B/R/C‐H microspheres exhibited tissue‐like morphology with active cells, indicating that sustained oxygen release and immunomodulation significantly supported cell viability and tissue morphology during long‐term culture.

Cell viability within the microspheres was analyzed using live/dead staining (Figure 9b). Significant differences in cell viability between the two groups were observed as early as day 7. Over time, the cell survival rate in the Sil@B group declined, with extensive cell death by day 28. In contrast, the Sil@B/R/C‐H group maintained high cell viability throughout the culture period, peaking in activity on day 21. These findings suggest that oxygen‐releasing immunomodulatory microspheres mitigate hypoxic conditions, providing sustained metabolic support and extending cell survival. Quantitative analysis (Figure 9e) confirmed that the Sil@B/R/C‐H group exhibited significantly higher cell survival areas compared to the Sil@B group during the long‐term culture period.

To evaluate the osteogenic differentiation potential of the microspheres, ALP staining, and activity assays were performed (Figure 9c,f). ALP staining in the Sil@B group was weak and did not show significant enhancement over time. In contrast, the Sil@B/R/C‐H group exhibited strong ALP staining starting on day 14, with further intensification by day 28, indicating robust early osteogenic differentiation activity. Quantitative analysis of ALP activity revealed that the Sil@B/R/C‐H group peaked on day 21, significantly outperforming the Sil@B group. These results validate the synergistic effects of sustained oxygen release and immunomodulation in promoting osteogenic differentiation under long‐term culture conditions.

To assess mineralization capacity, ARS staining and mineralization analysis were conducted (Figure 9d,g). The Sil@B group exhibited minimal mineralization throughout the culture period, whereas the Sil@B/R/C‐H group showed significant mineral deposition starting on day 14, with extensive calcium accumulation by day 28. Quantitative analysis of ARS staining indicated that the Sil@B/R/C‐H group significantly outperformed the Sil@B group in mineralization capacity, with the highest mineralization observed on day 28. These results demonstrate that oxygen‐releasing immunomodulatory microspheres not only support early osteogenic differentiation but also facilitate the formation of mineralized matrix, thus enabling the development of functional bone organoid units.

This study validates the potential of oxygen‐releasing immunomodulatory microspheres to generate functional bone organoid units through long‐term in vitro culture. The results demonstrated that Sil@B/R/C‐H microspheres exhibited peak cell activity on day 21, followed by significantly enhanced osteogenic differentiation and mineralization by day 28, leading to the formation of mature bone organoid units. These findings further confirm the mechanisms proposed in Sections 2.3 and 2.4, highlighting the synergistic effects of sustained oxygen release and immunomodulation in promoting bone tissue regeneration. From an immunological perspective, RAW264.7 macrophages polarize toward the anti‐inflammatory M2 phenotype, modulating inflammatory responses and creating a supportive immune environment for bone tissue regeneration.^[^ ^31^ ^]^ From an oxygenation perspective, sustained oxygen release from CaO₂‐HAp alleviates hypoxia within the microspheres, supporting BMSC proliferation and osteogenic differentiation, ultimately enhancing the functionality of bone organoid units.

This study not only demonstrates the feasibility of constructing bone organoid units under long‐term in vitro culture conditions using oxygen‐releasing immunomodulatory microspheres but also provides a new approach to bone regeneration. By combining immunomodulation with sustained oxygen supply, these microspheres establish a reliable foundation for the creation of bioactive, conformal bone organoid units, offering new possibilities for the treatment of critical‐sized bone defects and complex bone injuries.

### 2.7 Functional Validation of Bone Organoid Units In Vivo

Building upon the results of long‐term in vitro culture and in vivo bone repair experiments, this section further explores the performance of bone organoid units both in vitro and after in vivo transplantation. By transplanting bone organoid units, cultured for 28 days in vitro, into a critical‐sized cranial defect model in mice, this study comprehensively evaluated their potential for bone regeneration, angiogenesis, and immune modulation. The study highlights the mechanisms by which oxygen‐releasing immunomodulatory microspheres promote the construction and functional repair capabilities of bone organoid units.

**Figure 10:**
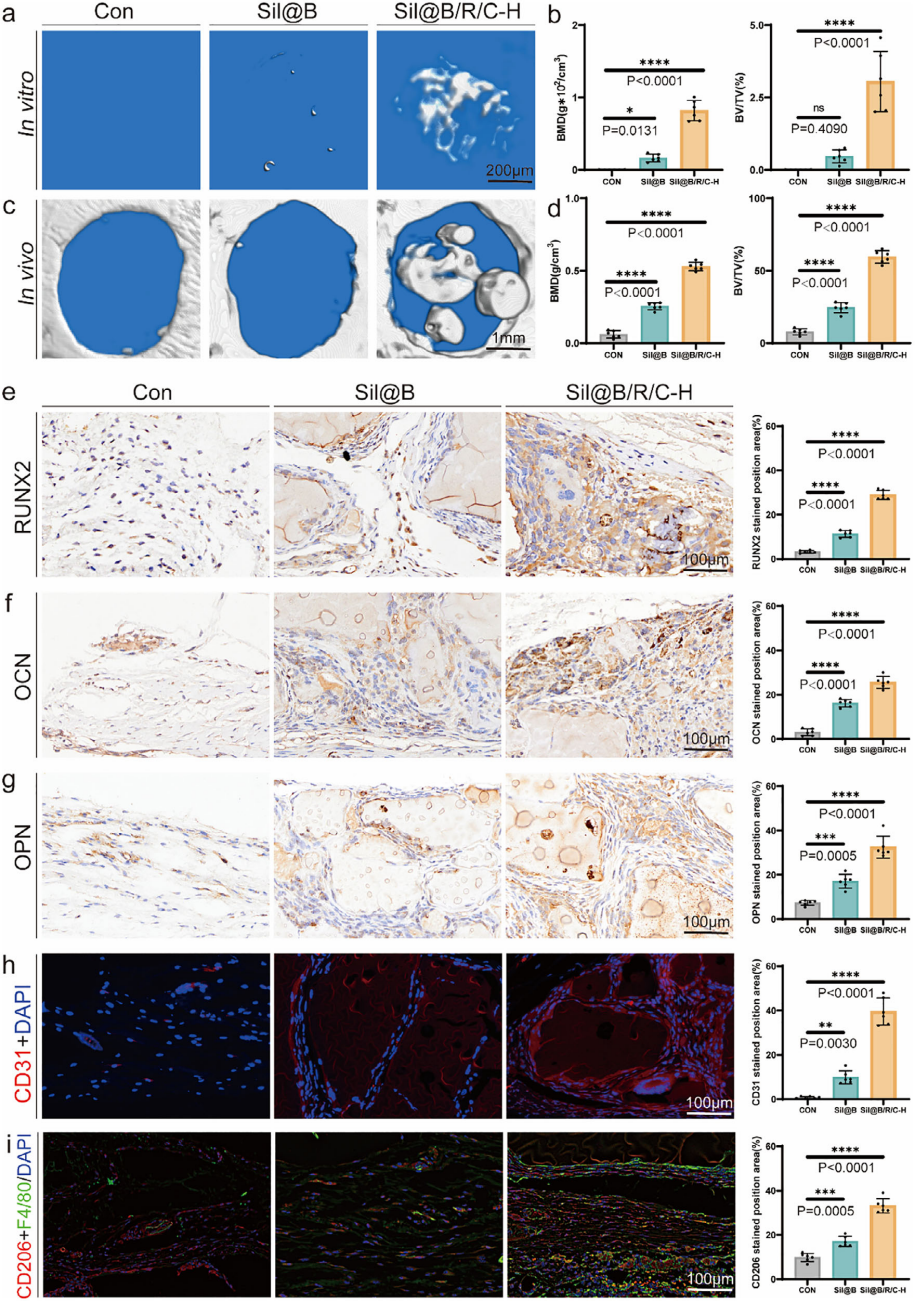
Functional Validation of Bone Organoid Units In Vivo. a) CT analysis of in vitro‐cultured bone organoid units after 28 days. b) Quantitative analysis of BMD and BV/TV of the in vitro‐cultured bone organoid units. c) CT images of cranial defects 8 weeks after transplantation of in vitro‐cultured bone organoid units. d) Quantitative analysis of BMD and BV/TV of the cranial defect area post‐transplantation. Immunohistochemical staining for RUNX2 e), OCN f), and OPN g) in cranial defect sections 8 weeks post‐transplantation. h) Immunofluorescence staining of CD31 in cranial defect sections. i) Immunofluorescence staining of CD206 in cranial defect sections. Data are presented as means ± SD (*n* = 6). ^*^ *p* < 0.05, ^**^ *p* < 0.01, ^***^ *p* < 0.001, ^****^ *p* < 0.0001.

CT analysis of bone organoid units after 28 days of in vitro culture revealed significant mineralization and dense matrix formation in the Sil@B/R/C‐H group (**Figure** **10**a). Compared to the Sil@B group, the Sil@B/R/C‐H group demonstrated a substantially higher degree of mineral deposition with uniform distribution. Quantitative analysis confirmed that BMD and bone volume‐to‐total volume ratio (BV/TV) were significantly higher in the Sil@B/R/C‐H group than in the Sil@B group (Figure 10b). These results demonstrate that the oxygen‐releasing immunomodulatory microspheres effectively enhance mineralization and provide a robust platform for the construction of bone organoid units in vitro.

To validate the functionality of in vitro‐cultured bone organoid units in vivo, organoid units from the Sil@B/R/C‐H and Sil@B groups were transplanted into critical‐sized cranial defects in mice, with the injection of SilMA microspheres serving as the control group (Con) (Figure 10c). Eight weeks post‐transplantation, CT analysis demonstrated minimal new bone formation in the Con group, with the defect remaining largely unhealed. The Sil@B group exhibited limited bone formation, while the Sil@B/R/C‐H group showed substantial defect repair, with dense and well‐organized bone tissue fully bridging the defect. Quantitative analysis further confirmed significantly higher BMD and BV/TV values in the Sil@B/R/C‐H group compared to the other groups (Figure 10d), highlighting the superior regenerative potential of the bone organoid units cultivated with oxygen‐releasing immunomodulatory microspheres.

The histological evaluation further revealed the differences in bone formation among the groups (Figure 10e–g). Immunohistochemical staining showed significantly higher expression of RUNX2, OCN, and OPN in the Sil@B/R/C‐H group compared to the Sil@B and Con groups. Quantitative analysis indicated that the positive staining areas for RUNX2, OCN, and OPN were significantly larger in the Sil@B/R/C‐H group (*p* < 0.0001), confirming the enhanced osteogenic differentiation and mineralization potential of the Sil@B/R/C‐H microspheres both in vitro and in vivo.

Angiogenesis and immune modulation are critical factors for successful bone defect repair.^[^ ^32^ ^]^ Immunofluorescence staining revealed significantly higher expression of CD31 and CD206 in the Sil@B/R/C‐H group compared to the Sil@B and Con groups (Figure 10h,i). Positive CD31 staining demonstrated that the Sil@B/R/C‐H group facilitated the formation of a dense vascular network within the defect area, providing essential nutrients and oxygen for tissue repair. Similarly, the increased CD206 expression indicated effective polarization of macrophages toward the anti‐inflammatory M2 phenotype, creating a favorable immune microenvironment for bone regeneration. Quantitative analysis confirmed that the CD31 and CD206‐positive staining areas were significantly larger in the Sil@B/R/C‐H group, underscoring the multifunctional advantages of oxygen‐releasing immunomodulatory microspheres in promoting vascularization and immune regulation.

This section validates the functional superiority of oxygen‐releasing immunomodulatory microspheres in constructing bone organoid units and repairing cranial bone defects. The Sil@B/R/C‐H group demonstrated excellent mineralization during in vitro long‐term culture and showed superior performance in bone regeneration, angiogenesis, and immune modulation after in vivo transplantation. These findings highlight the critical roles of sustained oxygen delivery and immune regulation in enhancing the functionality of bone organoid units. Taken together with the results from previous sections, this study systematically demonstrates the effectiveness of oxygen‐releasing immunomodulatory microspheres in facilitating both in vitro and in vivo bone regeneration. By addressing the challenges of hypoxia and immune imbalance, these microspheres provide a powerful and versatile platform for constructing bone organoid units and repairing complex bone defects. This innovative strategy offers promising insights and practical applications for personalized bone regeneration therapies and advanced bone tissue engineering, paving the way for future clinical translation.

## 3 Conclusion

This study presents the innovative design and application of silk fibroin hydrogel microspheres with sustained oxygen release and immunomodulatory functions, successfully constructing conformal bone organoid units and systematically evaluating their role and mechanisms in bone regeneration. To achieve a biomimetic and functional organoid microenvironment, BMSCs were incorporated as the primary osteogenic component due to their well‐established differentiation potential. RAW264.7 macrophages were co‐encapsulated to provide immunoregulatory functionality, particularly through their polarization toward the M2 phenotype, which promotes anti‐inflammatory signaling and supports tissue regeneration. CaO₂‐HAp nanoparticles served as the core oxygen‐releasing system, not only alleviating hypoxia but also contributing calcium and phosphate ions for mineralization. Catalase was introduced to decompose the intermediate hydrogen peroxide, thereby enhancing biocompatibility and preventing oxidative stress. This rational integration of cellular and material components enables the microspheres to simultaneously address hypoxia, inflammation, and osteogenesis—three major challenges in bone organoid engineering. The findings demonstrate that these microspheres effectively address the challenges of insufficient oxygen supply and impaired immune microenvironment regulation in bone regeneration. By continuously releasing oxygen to alleviate hypoxia and leveraging the immunomodulatory effects of RAW264.7 macrophages, the microspheres create a regenerative microenvironment conducive to osteogenic differentiation and angiogenesis. In vitro experiments validated the significant advantages of the oxygen‐releasing immunomodulatory microspheres in promoting cell viability, early osteogenic differentiation, and late‐stage mineralization. Results from an in vivo mouse cranial defect model further confirmed that these microspheres significantly enhance bone formation efficiency, improve vascularization, and optimize immune regulation. Notably, under long‐term culture conditions, these microspheres demonstrated the ability to form functional bone organoid units at the single‐microsphere level, offering new possibilities for the repair of complex bone defects.

Despite these promising outcomes, several limitations and challenges must be acknowledged. First, the scalability of the microsphere‐based bone organoid system for large‐scale production and clinical translation remains to be further validated. Second, while the current in vivo experiments in mice provide preliminary insights, the long‐term stability, biodegradation kinetics, and immune responses in larger animal models are still unknown and warrant further investigation. Additionally, the integration of bone organoid units into irregular and load‐bearing defect sites under mechanical stress presents another technical barrier. Addressing these challenges will require further refinement of fabrication methods, optimization of material compositions, and the use of more clinically relevant models. Moreover, broader accessibility and standardization of the fabrication platform will be essential to promote knowledge sharing and facilitate reproducibility across research groups.

The outcomes of this study not only provide an innovative solution for the treatment of critical‐sized bone defects but also lay a theoretical and practical foundation for the construction of vascularized bone organoids. In the future, with further optimization of bone organoid construction techniques, oxygen‐releasing immunomodulatory bone organoid units are expected to find widespread applications in bone tissue engineering and regenerative medicine. They hold the potential to serve as a novel platform for personalized and transplantable bone repair, paving the way for transformative advances in clinical bone regeneration therapies.

## 4 Experimental Section

### Materials

SilMA, LAP (Engineering for Life, Jiangsu). Liquid paraffin, Span 80 (Aladdin Shanghai). Microfluidic Coaxial needles (Nuokang Environmental Protection Technology, Qingdao). Ultra‐low adsorption culture plate (NEST, Wuxi). Fetal bovine serum (Sigma, Shanghai). Automatic cell counter (Countstar, Shanghai).

### Preparation of CaO_2_, CaO_2_‐HAp

To prepare CaO₂, dissolve 3 g of calcium chloride (CaCl₂) in water to create a 10% calcium chloride solution. Add 15 mL of ammonia solution (25–28%, 1 m) and 120 mL of PEG 200 to the solution. Subsequently, add 15 mL of hydrogen peroxide (H₂O₂) solution at a constant rate of 0.2 mL min^−1^ to the mixture while stirring continuously for 2 h. During the precipitation process, adjust the pH to 11.5 by adding 0.1 m sodium hydroxide (NaOH) solution. The resulting yellow precipitate is collected by centrifugation and washed three times with 0.4 m NaOH solution, followed by three washes with double‐distilled water (DDW) until the pH reaches 10. Finally, the obtained precipitate is dried in an oven at 80 °C, yielding CaO₂.^[^ ^33^ ^]^

To prepare CaO₂‐HAp, the synthesized CaO₂ is added to an appropriate amount of 100 mm PBS and stirred for 8 h. During this process, CaO₂ reacts with water to form Ca(OH)₂, which subsequently reacts with H₃PO₄ in PBS to form hydroxyapatite (Ca₁₀(PO₄)₆(OH)₂, HAp). Continuous stirring promotes the gradual deposition of HAp onto the surface of CaO₂ particles, resulting in a coated structure that enables sustained oxygen release.^[^ ^34^ ^]^

(1) $$10Ca{\left(OH\right)}_{2}+6H_{3}PO_{4}\to Ca_{10}{\left(PO_{4}\right)}_{6}{\left(OH\right)}_{2}+18H_{2}O$$

### Preparation of SilMA Microspheres and Oxygen‐Releasing Hydrogel Microspheres

Before preparing the hydrogel microspheres, the aqueous and oil phases were first prepared. In this study, the aqueous phase consisted of 15% SilMA and 0.05% ALP. For groups containing oxygen‐releasing components, CaO₂ or CaO₂‐HAp nanoparticles were first dispersed in PBS using a probe sonicator (5 s on/5 s off, 2 min total) to ensure uniform dispersion. The homogenized dispersion was then thoroughly mixed with the SilMA solution to form the functionalized aqueous phase. While the oil phase is composed of 90% paraffin oil and 10% Span 80.^[^ ^35^ ^]^

Microfluidic technology was utilized to fabricate the hydrogel microspheres, using a coaxial microfluidic needle setup, which includes two concentric needles. The inner needle delivers the aqueous phase, and the outer needle delivers the oil phase. The generation of microspheres relies on the shear force produced by the oil phase, which encapsulates the aqueous droplets in the oil, forming microspheres with uniform size. By adjusting the flow rate ratio of the oil and aqueous phases, the size of the microspheres can be precisely controlled. Under UV light at 365 nm, ALP in the aqueous phase undergoes photochemical crosslinking with SilMA, resulting in loosely porous hydrogel microspheres.

### Characterization of Oxygen‐Releasing Material, SilMA Microspheres and Oxygen‐Releasing Hydrogel Microspheres

The Scanning Electron Microscopy (SEM) characterization of CaO₂ and CaO₂‐HAp was conducted by dispersing nanoparticles in anhydrous ethanol, dropping onto silicon wafers, and sputter‐coating with a conductive layer. The SilMA microspheres and oxygen‐releasing hydrogel microspheres were freeze‐dried, adhered to conductive tape, and metal‐coated for SEM analysis. SEM (JSM‐7500F, Japan) was used to visualize the surface morphology. To confirm the incorporation and spatial distribution of the oxygen‐releasing components, energy‐dispersive X‐ray spectroscopy (EDX) was performed using the SEM system. EDX mapping of the SilMA@CaO₂ and SilMA@CaO₂‐HAp microspheres revealed uniform elemental distributions of calcium (Ca) and phosphorus (P), indicating successful and homogeneous embedding of CaO₂ and CaO₂‐HAp nanoparticles within the hydrogel network. This verified the structural integrity and functional uniformity of the oxygen‐releasing microspheres.

### Oxygen Release Measurement

Samples of CaO₂, CaO₂‐HAp, and catalase were placed in an anaerobic incubator and left undisturbed for one day to eliminate interference from residual oxygen. Similarly, pure water was deoxygenated by bubbling nitrogen gas through it, and then stored in the anaerobic incubator for one day. Subsequently, 150 mg of CaO₂ or 150 mg of CaO₂‐HAp were added to 30 mL of deoxygenated pure water containing catalase at a concentration of 1500 U mL^−1^. Deoxygenated pure water was used as the blank control. The dissolved oxygen levels in the three groups were measured under anaerobic conditions at 1, 3, 5, 7, 14, and 28 days using a dissolved oxygen meter (AR8606, Smart Sensor).

(2) $$CaO_{2}+2H_{2}O\to Ca{\left(OH\right)}_{2}+H_{2}O_{2}$$

(3) $$2H_{2}O_{2}\to O_{2}+2H_{2}O$$

### Characterization of SilMA Microspheres Properties—Swelling Test

The prepared hydrogel microspheres were immersed in PBS and incubated at 37 °C. The dry weight of the hydrogel microspheres was recorded as W_0_. At specific time intervals, the microspheres were removed from the incubator, their surfaces were gently dried, and their wet weight (W_t_ ​) was recorded. The swelling ratio was calculated using the following formula:

(4) $$\mathrm{Swelling}\;\mathrm{ratio}\left(\%\right)=\frac{\left(W_{t}-W_{0}\right)}{W_{0}}\times 100\%$$

### Degradation Test

The prepared hydrogel microspheres were freeze‐dried, and their initial dry weight was recorded as W_0_​. The microspheres were then immersed in PBS, DMEM medium, or a solution containing collagenase II, with the soaking solution replaced every three days. At specific time intervals, the microspheres were removed, and dried, and their dry weight (W_t_) was recorded. The degradation rate was calculated using the following formula:

(5) $$\mathrm{Degradation}\;\mathrm{ratio}\left(\%\right)=\frac{\left(W_{0}-W_{t}\right)}{W_{0}}\times 100\%$$

### Microsphere Mechanical Testing

To evaluate the mechanical properties of the hydrogel microspheres, single‐microsphere compression tests were performed in PBS using a micro‐force testing platform. A custom stainless‐steel flat‐end probe (diameter = 0.56 mm) equipped with a 3 × 3 mm platen was used to apply vertical compressive force onto individual microspheres. During the test, each microsphere was submerged in PBS and compressed from its full height (100%) to 75% of its original height. A high‐sensitivity force sensor continuously recorded the real‐time force and displacement at the probe tip. The resulting force–time, displacement–time, and force–displacement curves were analyzed. Gaussian fitting was performed using GraphPad Prism 9 software to characterize the mechanical deformation behavior of the microspheres.

### Preparation of SilMA@B, SilMA@B/R, SilMA@B/R/C and SilMA@B/R/C‐H

SilMA@B: For this group, 1 mL of 15% SilMA hydrogel was prepared, and 1 × 10^7^ BMSCs were resuspended in the hydrogel. The cell suspension was carefully mixed with the hydrogel to ensure uniform distribution of cells. The mixture was then processed using the microfluidic technique to generate the hydrogel microspheres. SilMA@B/R: In this group, 1 mL of 15% SilMA hydrogel was prepared, and a mixture of 8 × 10^6^ BMSCs and 2 × 10^6^ RAW 264.7 cells was resuspended in the hydrogel. The cells were thoroughly mixed with the hydrogel to ensure a homogeneous suspension. The final mixture was processed through the microfluidic system to generate uniform hydrogel microspheres. SilMA@B/R/C: For this group, 1 mL of 15% SilMA hydrogel was prepared, and a mixture of 8 × 10^6^ BMSCs, 2 × 10^6^ RAW 264.7 cells, and 1 mg of CaO_2_ was added. Additionally, 1000 U of catalase enzyme is incorporated into the mixture. The components were well‐mixed to ensure uniform distribution before being processed using the microfluidic system to generate the hydrogel microspheres. SilMA@B/R/C‐H: In this group, 1 mL of 15% SilMA hydrogel is prepared, and a mixture of 8 × 10^6^ BMSCs, 2 × 10^6^ RAW 264.7 cells, 1 mg of CaO_2_‐HAp, and 1000 U of catalase enzyme was added. The components are mixed thoroughly to achieve a homogeneous suspension. This mixture was then processed using the microfluidic system to produce the desired hydrogel microspheres.

The composition of the engineered microspheres, including the BMSCs to RAW264.7 cell ratio (4:1), the concentration of CaO₂ or CaO₂‐HAp (1 mg mL^−1^), and the dosage of catalase (1000 U mL^−1^), was determined based on the previous study,^[^ ^36^ ^]^ which demonstrated that this combination effectively promoted osteogenesis, immune modulation, and biocompatibility. In addition, the rational selection of oxygen‐releasing components and catalase was supported by the work of Daisuke Tomioka et al.,^[^ ^34^ ^]^ in which a similar oxygen delivery strategy significantly improved functional performance and cellular safety. Therefore, the current formulation adopted in this study builds upon proven effective designs and ensures a balance between oxygen supply, immunoregulation, and osteogenic potential for optimal bone organoid construction.

### Cytotoxicity and Proliferation Assay of Oxygen‐Releasing Microspheres

Hydrogel microspheres from the four experimental groups were stained using a live/dead cell double‐staining kit after culturing under hypoxic conditions for 1 day followed by normoxic conditions for 2 days. The microspheres were incubated in the dark for 30 min and then imaged using a confocal microscope (FV3000, Olympus, Japan). Cell viability for the four groups was assessed using the CCK‐8 assay. After incubating the samples for 2 h at a constant temperature, absorbance was measured at a wavelength of 450 nm.

### ALP Staining and ARS Staining with Quantitative Analysis of Mineralization in Oxygen‐Releasing Microspheres

To evaluate the osteogenic differentiation capacity of cells within the microspheres, an ALP staining assay was performed. Microspheres were fixed with 4% paraformaldehyde at room temperature for 15 min, washed with PBS, and incubated with BCIP/NBT ALP staining (Beyotime) solution at 37 °C for 30 min. After color development, images were captured using a stereomicroscope. ALP staining results were quantitatively analyzed using ImageJ software to calculate the percentage of stained areas, providing an assessment of ALP activity for the different groups of microspheres.

To assess the formation of mineralized matrices within the microspheres, an ARS staining assay was conducted. Microspheres were fixed with 4% paraformaldehyde for 20 min, followed by staining with 2% alizarin red S solution (pH 4.2) at room temperature for 20 min. After staining, the microspheres were washed with distilled water, observed, and imaged using a stereomicroscope. The ARS staining results were quantitatively analyzed using ImageJ software to calculate the percentage of stained areas, serving as an indicator of the mineralization capacity of the microspheres.

To quantitatively assess osteogenic activity and mineralization levels among different experimental groups, ALP and ARS staining results were subjected to image‐based analysis. Each experiment was performed with three biological replicates (*n* = 3). After staining, images of each group were acquired under consistent parameters using a microplate reader. The captured images were analyzed using ImageJ software to extract mean gray values or staining intensity as quantitative indicators. During analysis, unified thresholding and background correction were applied across all images. Statistical analysis was performed using GraphPad Prism software, and group differences were evaluated by one‐way ANOVA. A p‐value < 0.05 was considered statistically significant.

### qRT‐PCR Analysis of Gene Expression in Oxygen‐Releasing Microspheres

Quantitative real‐time PCR (qRT‐PCR) was performed to analyze the gene expression levels of inflammation‐, angiogenesis‐, and osteogenesis‐related markers in cells encapsulated within the microspheres. Total RNA was extracted using TRIzol reagent and reverse‐transcribed into cDNA using a reverse transcription kit. Gene amplification was carried out on a real‐time PCR instrument using SYBR Green dye. The relative expression levels of key markers, including TNF‐α, iNOS, CD86, IL‐10, CD206, VEGF, CD31, BMP‐2, RUNX2, and COL‐1, were normalized to the internal control CEL‐MIR‐39‐3P and calculated using the 2^−ΔΔCt method. All experiments were conducted in triplicate. The primer sequences required in the experiment are shown in Table S1 (Supporting Information).

### Implantation of Oxygen‐Releasing Microspheres in a Mouse Calvarial 3 mm Defect Model

The animal procedures adhered to the Guidelines for Care and Use of Laboratory Animals of Shanghai University and received approval from the University's Animal Ethics Committee (YS 2023–160). A critical‐sized calvarial defect model was established using 4–5‐week‐old C57BL/6 male mice (*n* = 6 per group). Under general anesthesia and sterile conditions, a circular defect with a diameter of 3 mm was created on the calvarial bone at the top of the skull. Microspheres from the following groups were implanted into the defect site: control group (Con, no treatment), Sil@B group, Sil@B/R group, Sil@B/R/C group, and Sil@B/R/C‐H group. After implantation, the defect area was sutured, and the mice were monitored postoperatively to ensure their health. Mice were sacrificed at 4‐ and 8‐weeks postsurgery for further analysis.

### Micro‐CT Analysis of Oxygen‐Releasing Microspheres

At 4‐ and 8‐weeks postsurgery, bone regeneration in the defect area was evaluated using micro‐computed tomography (micro‐CT). Samples were scanned at a resolution of 10 µm, and 3D reconstructions were performed to observe new bone formation at the defect site. Quantitative analysis of BMD, BV/TV, and Tb. N was conducted using micro‐CT analysis software.

### Histological Analysis of Oxygen‐Releasing Microspheres

The excised calvarial samples were subjected to histological analysis. Samples were fixed in 4% paraformaldehyde, decalcified, dehydrated, embedded in paraffin, and sectioned at a thickness of 5 µm. Sections were stained with hematoxylin and eosin (H&E) to observe tissue structure and with Masson's trichrome stain to assess collagen deposition. The stained sections were examined under a light microscope to evaluate bone regeneration and ECM remodeling.

### Immunohistochemical Analysis of Oxygen‐Releasing Microspheres

Immunohistochemical staining was performed to assess the expression of osteogenesis‐related markers in the post‐surgery samples, including OCN, OPN, and RUNX2. Paraffin sections were deparaffinized, rehydrated, and subjected to antigen retrieval. The sections were incubated with primary antibodies against OCN, OPN, and RUNX2, followed by incubation with horseradish peroxidase (HRP)‐conjugated secondary antibodies. Staining was visualized using a DAB substrate, and hematoxylin was used for counterstaining. The stained sections were imaged using a light microscope, and the percentage of positive staining areas was quantitatively analyzed using ImageJ software.

### Immunofluorescence Analysis of Oxygen‐Releasing Microspheres

Immunofluorescence staining was conducted to evaluate the role of microspheres in promoting angiogenesis and immunomodulation. Key markers included CD31 and CD206. Frozen tissue sections were prepared and blocked, followed by incubation with primary antibodies against CD31 and CD206. After incubation with fluorescence‐conjugated secondary antibodies, the sections were counterstained with DAPI to label nuclei. Fluorescent images were captured using a fluorescence microscope, and ImageJ software was used to quantify the percentage of CD31‐ and CD206‐positive staining areas.

### In Vivo Biosafety Evaluation

To assess systemic biocompatibility, major organs—including the heart, liver, spleen, lung, and kidney—were collected from mice in each treatment group (Con, Sil@B, Sil@B/R, Sil@B/R/C, and Sil@B/R/C‐H) after 8 weeks of in vivo implantation. The harvested tissues were rinsed with PBS, fixed in 4% paraformaldehyde for 24 h, dehydrated, embedded in paraffin, and sectioned into 5 µm‐thick slices. The sections were stained with H&E according to standard protocols. Histological observations were performed using an optical microscope to assess tissue architecture, inflammatory infiltration, necrosis, or other pathological changes.

### Subcutaneous Implantation of Oxygen‐Releasing Microspheres for Bone Organoid Formation

Male BALB/c nude mice (4–5 weeks old) were used to evaluate the formation of bone organoids through subcutaneous implantation of oxygen‐releasing microspheres (*n* = 6). Under sterile conditions, microspheres from different experimental groups (Con, Sil@B, Sil@B/R, Sil@B/R/C, and Sil@B/R/C‐H) were implanted subcutaneously into the dorsal region of each mouse. The mice were maintained under normal conditions post‐surgery and sacrificed at 8 weeks to analyze the morphological, histological, and immunological characteristics of the bone organoids.

### Histological, Immunohistochemical, and Immunofluorescence Analysis of Bone Organoids

The retrieved bone organoid samples were fixed in 4% paraformaldehyde, dehydrated, and embedded in paraffin. H&E staining was performed to observe tissue structure, and Masson's trichrome staining was used to assess collagen deposition. Microscopic images were captured to analyze tissue structure and ECM remodeling in the organoids. Immunohistochemical staining for OPN and OCN and immunofluorescence staining for CD31and CD206 were performed to evaluate new bone formation, vascularization, and inflammatory responses. The percentage of stained areas was quantitatively analyzed using ImageJ software.

### Long‐Term In Vitro Culture and Osteogenic Differentiation Validation of Bone Organoid Units

To assess the role of oxygen‐releasing microspheres in constructing bone organoid units, Sil@B and Sil@B/R/C‐H microspheres were cultured long‐term under standard cell culture conditions (37 °C, 5% CO₂) using ultra‐low adhesion culture plates to prevent substrate attachment. For the first 3 days, microspheres were maintained in α‐MEM medium supplemented with 10% fetal bovine serum and 1% penicillin‐streptomycin. After three days, the culture was switched to an osteogenic induction medium consisting of DMEM, 10% fetal bovine serum, 1% penicillin‐streptomycin, 10 mmol L^−1^ β‐glycerophosphate, 50 µg mL^−1^ ascorbic acid, and 10 nmol L^−1^ dexamethasone. Medium replacement was performed every 2 days during the first 7 days, daily from day 7 to 14, and twice daily from day 14 to 28.

The morphological changes and tissue formation of the microspheres were periodically observed during the culture period, with their morphology and thickening recorded under a microscope. To evaluate cell viability and survival rates, live/dead cell fluorescence staining was performed on microspheres at each time point. Microspheres were stained with Calcein‐AM and PI solutions to label live and dead cells, respectively, and observed under a fluorescence microscope to examine cell distribution. The live cell area was quantitatively analyzed using ImageJ software, and the cell activity was compared across different time points.

To assess the osteogenic differentiation potential of the microspheres, ALP staining was conducted on days 7, 14, 21, and 28. Microspheres were treated with ALP staining solution, and the stained regions were observed under a light microscope. The ALP‐stained area was quantitatively analyzed using ImageJ software to compare osteogenic differentiation between the two groups at different time points. To evaluate the formation of a mineralized matrix within the microspheres, ARS staining was performed at the end of the culture period. Microspheres were stained with ARS solution, excess dye was removed, and calcium deposition was observed under a microscope. The ARS‐stained area was quantitatively analyzed using ImageJ software to evaluate the mineralization capacity of microspheres at different time points.

### In Vivo Cranial Bone Repair and Functional Evaluation of Bone Organoid Units

The repair ability of bone organoid units was evaluated using a cranial critical‐sized defect model in male C57BL/6 mice (4‐5 weeks old), with circular defects of 3 mm in diameter created at the parietal bone region. Bone organoid units cultured in vitro for 28 days, including Sil@B/R/C‐H and Sil@B groups, were injected and implanted into the defect area, with a Con as a comparison. After implantation, the skin over the defect area was directly sutured, and the mice were maintained for 8 weeks. After harvesting, CT scans were performed to obtain images of bone repair at the defect site, and BMD and BV/TV were analyzed to assess the bone repair outcomes. Tissue sections were stained with immunohistochemistry to detect osteogenic markers, including RUNX2, OCN, and OPN, and the relative area of positive staining was quantified using ImageJ software to evaluate osteogenic differentiation and mineralization. Additionally, immunofluorescence staining was performed to detect angiogenic marker CD31 and immunoregulatory marker CD206 in the repair area, with quantitative analysis of the positive staining area.

### Statistical Analysis

All statistical analyses were performed using GraphPad Prism 9. Data are presented as mean ± standard deviation (SD). For comparisons between the two groups, unpaired two‐tailed Student's *t*‐tests were used. For comparisons involving three or more groups, one‐way analysis of variance (ANOVA) with Tukey's post‐hoc test was applied. Statistical significance was defined as ^*^ *p* < 0.05, ^**^ *p* < 0.01, ^***^ *p* < 0.001, and ^****^ *p* < 0.0001.

## Conflict of Interest

The authors declare no conflict of interest.

## Author Contributions

A.D., H.Z., Y.H., and J.L. contributed equally to this work. A.D. wrote, reviewed, and edited the final draft and wrote the original draft, methodology, and data curation. H.Z., J.W., and X.C. performed the methodology. Y.H. wrote the original draft. J.L. and Y.L. performed data curation. Z.G. and K.X. performed a formal analysis. J.W. and Y.J. performed supervision. L.B. performed supervision and conceptualization. J.S. performed supervision and funding acquisition.

## Supporting information



Supporting Information

## Data Availability

The data that support the findings of this study are available from the corresponding author upon reasonable request.
