# Supplementary material for: Microsphere Strategy to Generate Conformal Bone Organoid Units with Osteoimmunomodulation and Sustainable Oxygen Supply for Bone Regeneration
Source: Adv Sci (Weinh). 2025 Jun 20;12(32):e01437. doi: 10.1002/advs.202501437 (PMC12407368; doi:10.1002/advs.202501437)
Supplement: Supplementary file 1 — Supporting Information [file ADVS-12-e01437-s001.docx]

**Table S1.** Primer sequences for q-PCR.

| **Gene symbol** | **5’-3’** |
| --- | --- |
| TNF-α-F | CTGAACTTCGGGGTGATCGG |
| TNF-α-R | GGCTTGTCACTCGAATTTTGAGA |
| IL-1β-F | GAAATGCCACCTTTTGACAGTG |
| IL-1β-R | TGGATGCTCTCATCAGGACAG |
| TGF-β-F | CCACCTGCAAGACCATCGAC |
| TGF-β-R | CTGGCGAGCCTTAGTTTGGAC |
| Arg-1-F | CTCCAAGCCAAAGTCCTTAGAG |
| Arg-1-R | GGAGCTGTCATTAGGGACATCA |
| IL-6-F | CTGCAAGAGACTTCCATCCAG |
| IL-6-R | AGTGGTATAGACAGGTCTGTTGG |
| CD86-F | CTGCTCATCATTGTATGTCAC |
| CD86-R | ACTGCCTTCACTCTGCATTTG |
| IL-10-F | GAGAAGCATGGCCCAGAAATC |
| IL-10-R | GAGAAATCGATGACAGCGCC |
| CD163-F | GGCTAGACGAAGTCATCTGCAC |
| CD163-R | CTTCGTTGGTCAGCCTCAGAGA |
| iNOS-F | CACCAAGCTGAACTTGAGCG |
| iNOS-R | CGTGGCTTTGGGCTCCTC |
| CD31-F | AAGTGGAGTCCAGCCGCATATC |
| CD31-R | ATGGAGCAGGACAGGTTCAGTC |
| VEGF-F | TTGCCTTGCTGCTCTACCTCCA |
| VEGF-R | GATGGCAGTAGCTGCGCTGATA |
| RUNX2-F | CCTGAACTCTGCACCAAGTCCT |
| RUNX2-R | TCATCTGGCTCAGATAGGAGGG |
| BMP2-F | AGTAGTTTCCAGCACCGAATTA |
| BMP2-R | CACTAACCTGGTGTCCAATAGT |
| COL1-F | CCTCAGGGTATTGCTGGACAAC |
| COL1-R | CAGAAGGACCTTGTTTGCCAGG |


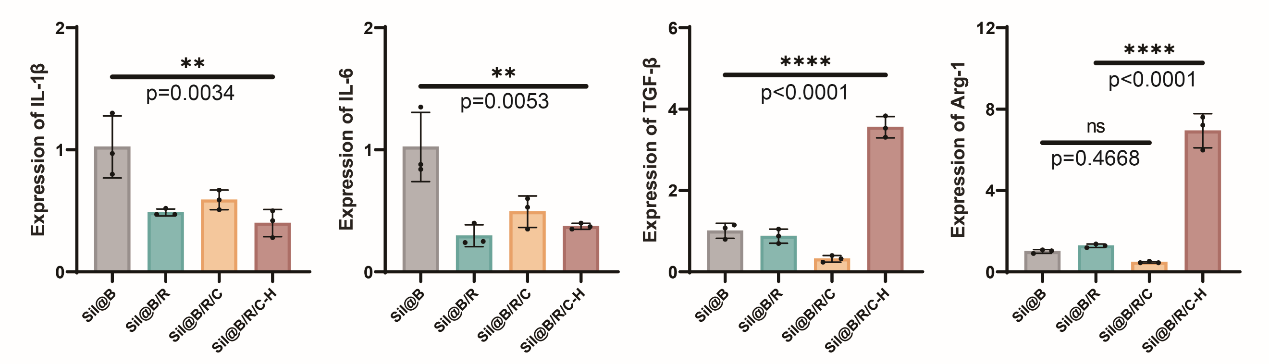


**Fig. S1. Immunoregulation of oxygen-releasing microspheres.** Gene expression analysis of inflammatory markers (IL-1β and IL-6), anti-inflammatory markers (TGF-β and Arg-1) (n=3).


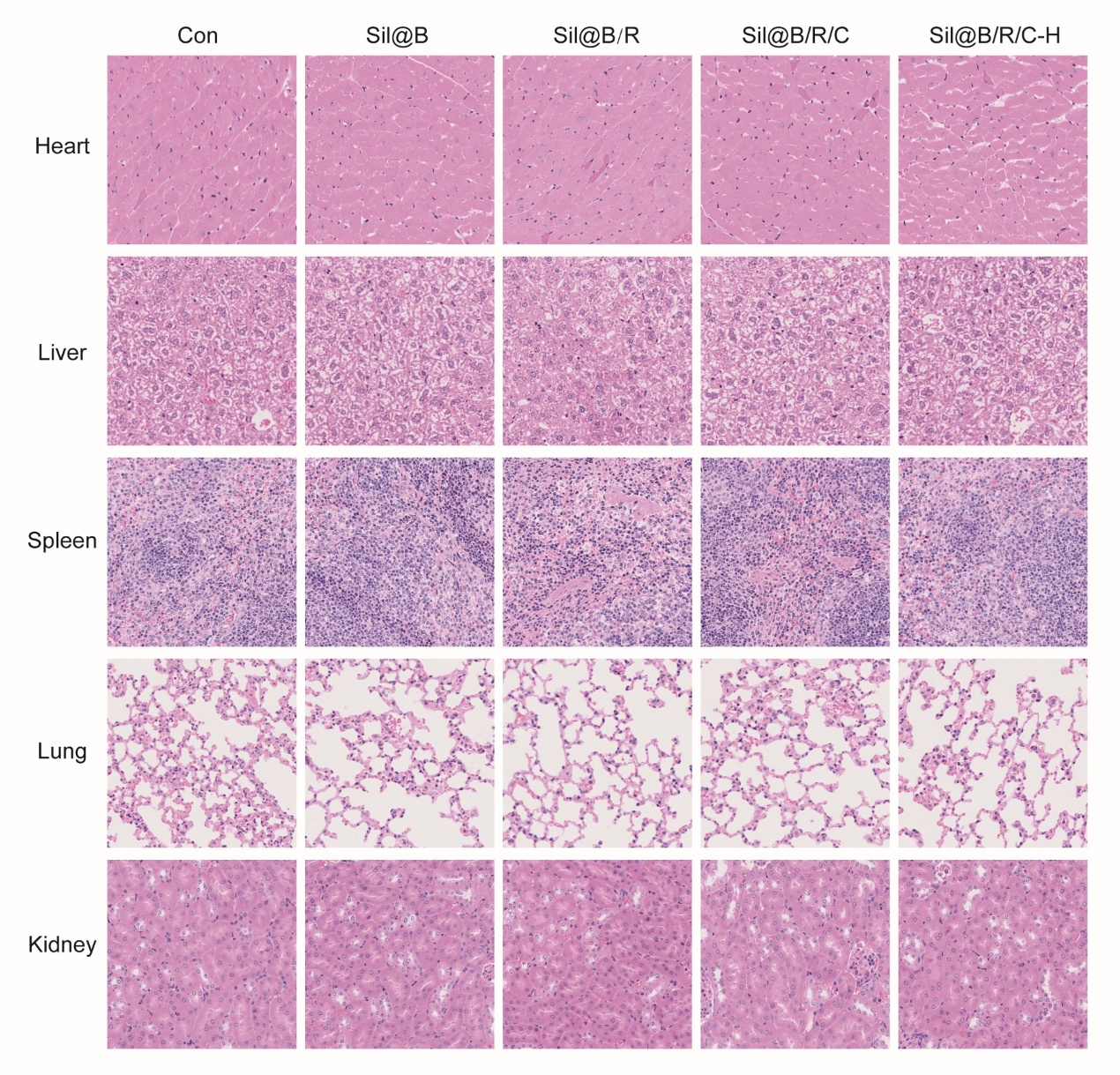


**Fig. S2. Histological evaluation of major organs after implantation of oxygen-releasing immunomodulatory microspheres.** Representative H&E-stained sections of the heart, liver, spleen, lung, and kidney collected 8 weeks after implantation in each group (Con, Sil@B, Sil@B/R, Sil@B/R/C, and Sil@B/R/C-H) (n=6).
